# Adult-onset CNS myelin sulfatide deficiency is sufficient to cause Alzheimer’s disease-like neuroinflammation and cognitive impairment

**DOI:** 10.1186/s13024-021-00488-7

**Published:** 2021-09-15

**Authors:** Shulan Qiu, Juan Pablo Palavicini, Jianing Wang, Nancy S. Gonzalez, Sijia He, Elizabeth Dustin, Cheng Zou, Lin Ding, Anindita Bhattacharjee, Candice E. Van Skike, Veronica Galvan, Jeffrey L. Dupree, Xianlin Han

**Affiliations:** 1 Barshop Institute for Longevity and Aging Studies, University of Texas Health Science Center at San Antonio, 4939 Charles Katz Drive, San Antonio, TX 78229 USA; 2 Division of Diabetes, Department of Medicine, University of Texas Health Science Center at San Antonio, San Antonio, TX 78229 USA; 3 Present Address: State Key Lab. of Environmental & Biological Analysis, Department of Chemistry, Hong Kong Baptist University, Hongkong, China; 4 Department of Anatomy and Neurobiology, Virginia Commonwealth University, Richmond, Virginia 23284 USA; 5 BRC Bioinformatics Facility, Institute of Biotechnology, Cornell University, Ithaca, NY 14853 USA; 6 College of Food Science and Engineering, Ocean University of China, Qingdao, 266003 China; 7 Department of Cellular and Integrative Physiology, University of Texas Health Science Center at San Antonio, San Antonio, TX 78229 USA; 8 Research Division, McGuire Veterans Affairs Medical Center, Richmond, Virginia 23249 USA

**Keywords:** Sulfatide, Alzheimer’s disease, Astrogliosis, Microgliosis, Neuroinflammation, Cognitive impairment, Lipidomics, RNA profiling, Cerebroside sulfotransferase (CST)

## Abstract

**Background:**

Human genetic association studies point to immune response and lipid metabolism, in addition to amyloid-beta (Aβ) and tau, as major pathways in Alzheimer’s disease (AD) etiology. Accumulating evidence suggests that chronic neuroinflammation, mainly mediated by microglia and astrocytes, plays a causative role in neurodegeneration in AD. Our group and others have reported early and dramatic losses of brain sulfatide in AD cases and animal models that are mediated by ApoE in an isoform-dependent manner and accelerated by Aβ accumulation. To date, it remains unclear if changes in specific brain lipids are sufficient to drive AD-related pathology.

**Methods:**

To study the consequences of CNS sulfatide deficiency and gain insights into the underlying mechanisms, we developed a novel mouse model of adult-onset myelin sulfatide deficiency, i.e., tamoxifen-inducible myelinating glia-specific cerebroside sulfotransferase (CST) conditional knockout mice (CST^fl/fl^/Plp1-CreERT), took advantage of constitutive CST knockout mice (CST^−/−^), and generated CST/ApoE double knockout mice (CST^−/−^/ApoE^−/−^), and assessed these mice using a broad range of methodologies including lipidomics, RNA profiling, behavioral testing, PLX3397-mediated microglia depletion, mass spectrometry (MS) imaging, immunofluorescence, electron microscopy, and Western blot.

**Results:**

We found that mild central nervous system (CNS) sulfatide losses within myelinating cells are sufficient to activate disease-associated microglia and astrocytes, and to increase the expression of AD risk genes (e.g., *Apoe, Trem2, Cd33,* and *Mmp12*), as well as previously established causal regulators of the immune/microglia network in late-onset AD (e.g., *Tyrobp, Dock,* and *Fcerg1*), leading to chronic AD-like neuroinflammation and mild cognitive impairment. Notably, neuroinflammation and mild cognitive impairment showed gender differences, being more pronounced in females than males. Subsequent mechanistic studies demonstrated that although CNS sulfatide losses led to ApoE upregulation, genetically-induced myelin sulfatide deficiency led to neuroinflammation independently of ApoE. These results, together with our previous studies (sulfatide deficiency in the context of AD is mediated by ApoE and accelerated by Aβ accumulation) placed both Aβ and ApoE upstream of sulfatide deficiency-induced neuroinflammation, and suggested a positive feedback loop where sulfatide losses may be amplified by increased ApoE expression. We also demonstrated that CNS sulfatide deficiency-induced astrogliosis and ApoE upregulation are not secondary to microgliosis, and that astrogliosis and microgliosis seem to be driven by activation of STAT3 and PU.1/Spi1 transcription factors, respectively.

**Conclusion:**

Our results strongly suggest that sulfatide deficiency is an important contributor and driver of neuroinflammation and mild cognitive impairment in AD pathology.

**Supplementary Information:**

The online version contains supplementary material available at 10.1186/s13024-021-00488-7.

## Introduction

AD is the most common cause of dementia in older individuals. However, effective disease-modifying therapies remain elusive [1], underscoring the need to understand better the molecular mechanism(s) underlying disease etiology. Glial responses in AD include molecular, morphological, and functional changes in astrocytes and microglia [2, 3]. Accumulating evidence has implicated sustained glia-mediated inflammation as a major contributor to AD neurodegenerative processes and cognitive deficits [4, 5]. Recent massive genome-wide association studies (GWAS) [6, 7] and next-generation sequencing [8–10] have convincingly associated more than 50 genes/loci with AD, most of which are involved in neuroinflammation/immune activation and are preferentially expressed in the brain by microglia. Similarly, integrative network-based analysis has implicated the immune/microglial network in late-onset AD [11]. In addition, recent single-cell transcriptomic studies have consistently revealed that microglia and astrocytes are among the cell types with the most significant gene expression changes in AD [12–14], displaying specific gene expression profiles described as disease-associated microglia (DAMs) [15] and disease-associated astrocytes (DAAs) [16]. IRF8 and STAT3 are critical transcription factors for transforming microglia and astrocyte into a reactive phenotype, respectively [17, 18], and PU.1/Spi1, another master regulator of myeloid cells that controls microglial development and function [19], is also a risk gene for AD [20].

It is well-established that ApoE ε4 allele remains the strongest genetic risk factor for AD and the ApoE ε2 allele the strongest genetic protective factor, and the understanding of ApoE pathogenesis has expanded beyond amyloid-β to tau neurofibrillary degeneration, microglia and astrocyte responses, and blood-brain barrier disruption [21, 22]. Meanwhile, ApoE is the major extracellular lipid carrier in the central nervous system (CNS) [23], where it is primarily produced by astrocytes and to a lower extent by microglia [24]. Besides revealing a critical role of neuroinflammation in AD, large-scale human studies have also consistently linked lipid metabolism-related genes with AD pathogenesis [6, 7]. Besides ApoE, many more recently identified AD susceptible loci/genes are also involved in lipid metabolism (e.g., *Clu, Plcg2, Abca7, Abca1, Trem2*) [6, 7].

The brain is the richest organ in terms of lipid content and diversity, largely due to the abundance of lipid-rich myelin [25]. Although AD is not generally considered or classified as a demyelinating disease, there is evidence of focal demyelination in AD patients and transgenic mouse models, which is related with Aβ and neurofibrillary pathology [26, 27]. Numerous studies have shown early and robust transcriptional changes in myelin and oligodendrocyte genes in AD [13, 28]. AD has even been described as homeostatic responses to age-related myelin breakdown [29]. Multi-dimensional mass spectrometry-based shotgun lipidomic studies from our laboratory have revealed that sulfatides, a class of sulfoglycolipids highly enriched in myelin, are specifically and dramatically reduced at the earliest clinically recognizable stages of AD, with sulfatide losses being particularly severe in gray matter, but also evident in white matter and cerebrospinal fluid [30–34]. Likewise, our group and others have reported significant losses of brain sulfatide levels in AD cases and animal models that strongly correlate with the onset and severity of amyloid beta (Aβ) deposition [35–40]. Multiple lines of evidence support a strong relationship between ApoE and sulfatide, including the observation that sulfatide accumulates in the brains of mice lacking ApoE [23] or sortilin (Sort1) [41], a novel ApoE receptor that has been implicated with AD [42, 43]. Moreover, mechanistic studies have revealed that sulfatide deficiency in AD occurs in an ApoE-dependent and isoform-specific manner [23, 35]. In addition, the gene that codes for the enzyme that degrades sulfatide (arylsulfatase A) was recently linked to AD [44, 45]; while TREM2, a well-established AD risk gene [46], has been shown to interact quite strongly with sulfatide [47].

To investigate the consequences and related molecular mechanisms of adult-onset sulfatide deficiency, a specific event that occurs very early in AD, on brain homeostasis and cognitive function, we generated a novel mouse model by inducible and conditional depletion of the cerebroside sulfotransferase (CST, a.k.a. Gal3st1) gene, which codes for the enzyme that catalyzes the last step of sulfatide biosynthesis, within myelinating cells. Strikingly, our results revealed for the first time that mild adult-onset CNS myelin sulfatide deficiency is sufficient to cause chronic AD-like neuroinflammation, characterized by strong activation of DAAs and DAMs, and mild cognitive impairment.

## Materials and methods

### Mice

The new Cst loxP/loxP (CST^fl/fl^) mouse model was generated using the Clustered Regularly Interspaced Short Palindromic Repeats (CRISPR) technology by Applied StemCell, Inc. (USA). Briefly, a mixture of active guide RNA molecules (gRNAs), two single-stranded oligodeoxynucleotides (ssODN) donors, and qualified Cas-9 mRNA was prepared and injected into the cytoplasm of C57BL/6 embryos. A SURVEYOR mutation detection assay was performed on four potential gRNAs according to the manufacturer’s instructions (Transgenomic Inc., Cat# 706020). Two gRNAs (indel frequency of 23 and 0%, and 13 and 0%) were selected for the generation of donor DNA and gRNA transcripts for microinjection. To generate the donor DNA based on the sequences of active gRNAs, ssODNs were synthesized to insert the LoxP sequence at the two designated sites of mGal3st1 gene flanking exons 3 and 4 (Fig. S1). The presence of LoxP sites at designated locations was confirmed by sequencing the modified regions in the mCst1 gene locus.

Then, CST^fl/fl^ mice were crossed with Plp1-CreERT^+^ mice (Stock No: 005975, the Jackson Laboratory, Bar Harbor, ME, USA). Tamoxifen (40–60 mg tamoxifen/kg body weight) was administered via intraperitoneal injection once every 24 h for a total of 4 consecutive days to CST^fl/fl^/Plp1-CreERT^−^ (CST Cre^−^) and CST^fl/fl^/Plp1-CreERT^+^ (CST Cre^+^) male and female mice.

The CST conditional knockout mouse line (CST cKO) and the CST constitutive knockout (CST KO) mouse line were both on a C57BL/6 J background and housed in groups of ≤5 mice/cage, maintained in a temperature- and humidity-controlled environment with a 12-h light-dark cycle. They were provided with food and water ad libitum. The protocols for animal experiments were conducted in accordance with the ‘Guide for the Care and Use of Laboratory Animals’ (8th edition, National Research Council of the National Academies, 2011) and were approved by the Animal Studies Committee of The University of Texas Health Science Center at San Antonio.

AIN-76A Rodent Diet (D1000) and pexidartinib (PLX3397) (C-1271, Chemgood, Glen Allen, VA, USA) containing diet (D15112401, 290 mg PLX3397/kg of AIN-76A diet) were prepared by Research Diets Inc. (New Brunswick, NJ, USA). CST^+/+^ and CST^−/−^ mice were fed with PLX3397-containing or control chow-like diet from 1 to 3 mo of age.

### Animal behavior

The measurement of cognitive function was done by the Integrated Physiology of Aging Core of San Antonio Nathan Shock Center. First, all animals were subjected to a battery of tests that included righting reflex, hindlimb clasping, crossed extensor reflex, forelimb/hindlimb placing responses, grasp reflex, rooting reflex, vibrissa placing response, negative geotaxis, and auricular startle tests. Mice had to pass this initial neurobehavioral screening for inclusion in subsequent studies. The second assessment was the frailty index [48]. The third was to assess cognitive function following Morris Water Maze (MWM) and Novel Object Recognition (NOR) paradigms.

The MWM paradigm, originally designed by Morris et al. [49], tests spatial learning and memory. Mice were given a series of 4 trials per day, 1 h apart, to locate a submerged (hidden) platform (1 cm below water surface) in a large tank (120 cm in diameter) filled with opaque white-colored water maintained at a temperature of 22.0 ± 1.0 °C. For each trial, the following parameters were analyzed using a computer-interface camera tracking system (TopScan Suite, CleverSys Inc., Reston, VA, USA): length of path to platform and latency to platform, swim velocity, percent of swim path limited to outer annulus, as well as percent of path in platform annulus (36-cm-diameter circular area surrounding platform). After 5 training days, a single 60 s probe trial was performed with the platform removed. Time in each quadrant and times passing through former target location were analyzed.

NOR was used to evaluate non-spatial recognition memory. The assay was performed over three consecutive days. Initially, mice were habituated individually to the open arenas for 5 min. For the training session, two matching objects were placed in the arena and the animal was allowed to explore for 5 min while the computer-interfaced camera tracking system (TopScan Suite, CleverSys Inc., Reston, VA, USA) recorded the amount of time the animal spent exploring the objects. On the novel object recognition test (24 h after the training session), one of the familiar objects was placed in the arena, along with a distinct, novel object the animal had never seen before. The time spent exploring each object was recorded for 5 min. A preference ratio was calculated based on the time spent with each object ((novel-familiar)/total object exploration), such that a positive number indicates the novel object is preferred and a negative number indicates the familiar object is preferred.

### Brain preparation

For histological analysis, mice were anesthetized with isoflurane and perfused with PBS. Right-brain hemispheres were fixed in 4% PFA overnight and placed in 10, 20, and 30% sucrose solution subsequently before freezing and cutting on a freezing sliding microtome. Serial 10 μm coronal sections of the brain or brain stem were collected, the hippocampus being used as a landmark. For biochemical and mRNA expression analysis, the left-brain hemispheres, brain stem, spinal cord, and sciatic nerve were dissected out and flash frozen in liquid nitrogen.

### Lipid extraction and mass spectrometric analysis of lipids

Multidimensional mass spectrometry-based shotgun lipidomics was performed as previously described [50]. Briefly, frozen brain, spinal cord, or sciatic nerve tissues were homogenized in ice-cold phosphate-buffered saline (PBS) using Precellys® Evolution Tissue Homogenizer (Bertin, France). The protein concentration of homogenates was determined using the Bio-Rad protein assay (Bio-Rad, Hercules, CA, USA). Lipids were extracted by a modified procedure of Bligh and Dyer extraction in the presence of internal standards, which were added based on the total protein content of each sample [51]. Lipids were assessed using a triple-quadrupole mass spectrometer (TSQ Altis, (Thermo Fisher Scientific, Waltham, MA, USA) equipped with a Nanomate device (Advion Ithaca, NY, USA) and Xcalibur system as previously described [52]. Data processing including ion peak selection, baseline correction, data transfer, peak intensity comparison, ^13^C deisotoping, and quantitation were conducted using a custom-programmed Microsoft Excel macro as previously described [53].

### Mass spectrometry imaging

As described previously [54], fresh frozen slices were transferred onto the same conductive side of indium tin oxide (ITO) slides. The matrix of N-(1-naphthyl) ethylenediamine dihydrochloride (NEDC) was applied by the Bruker ImagePrep device (Bruker Daltonics, Germany). MALDI mass spectra were acquired in the negative ion mode using a reflectron geometry MALDI-TOF mass spectrometer of Ultraflextreme (Bruker Daltonics, Germany) equipped with a neodymium-doped yttrium aluminum garnet (Nd:YAG)/355-nm laser as the excitation source. Imaging data were analyzed using FlexImaging v3.0 and FlexAnalysis v3.4. The intensity of lipids was demonstrated as a false-color image based on the mass spectrometry peak.

### Gene expression analysis

Brain or spinal cord tissue was frozen in liquid nitrogen and powdered, followed by RNA extraction using the Animal Tissue RNA Purification Kit (Norgen, Canada) according to manufacturer protocol. The concentration of RNA was determined using DS-11 Spectrophotometer (DeNovix, USA). Then multiplex gene expression analysis was performed using the NanoString nCounter® Technology with the Mouse Neuroinflammation Panel and nCounter® SPRINT™ Profiler according to the manufacturer protocol (NanoString Technologies, USA). The data were analyzed using nSolver 4.0 software. Our analysis included background subtraction using the mean of Negative Controls, standard normalization using Positive Control Normalization and CodeSet Content Normalization. Real time qPCR was performed using 7900HT Fast Real-Time PCR System (Applied Biosystems, USA) with SYBR Green Master Mix (Applied Biosystems by Thermo Fisher Scientific, USA). Primers for mouse are Gfap (Forward 5’ACCGCATCACCATTCCTGTAC3’, Reverse 5’TGGCCTTCTGACACGGATTT3’), Cd68 (Forward5 ‘TGTCTGATCTTGCTAGGACCG3’, Reverse 5’GAGAGTAACGGCCTTTTTGTGA3’), Iba1 (Forward 5’GGATTTGCAGGGAGGAAAAG3’, Reverse 5′ TGGGATCATCGAGGAATTG3’), and Cd11b (Forward 5’GTGTGACTACAGCACAAGCCG3’, Reverse 5’CCCAAGGACATATTCACAGCCT3’). Melting curve analysis was performed at the end of each PCR reaction. Relative gene expression was calculated after normalization by a housekeeping gene (GAPDH).

### Western blotting

Brain, spinal cord or brain stem tissue was frozen in liquid nitrogen and powdered, followed by homogenization in 1xNP40 lysis buffer with Halt Protease and Phosphatase Inhibitor Cocktails (Thermo Scientific) using Precellys® Evolution Tissue Homogenizer (Bertin, France). Homogenates were centrifuged at 12,000 g for 30 min at 4 °C; protein concentration of supernatants was determined using the Bio-Rad protein assay (Bio-Rad, Hercules, CA, USA). Supernatants were run with NuPage 4–12% Bis-Tris gels (Life Technologies, Grand Island, NY, USA) or 10% gels (Bio-Rad, USA) under reducing conditions. The PVDF (Fluor) membranes (CliniSciences, France) with the transferred protein were incubated with primary antibodies (1:1000–2000 dilution) of anti-GFAP (chicken, Millipore, USA), anti-Iba1 (Rabbit, FUJIFILM Wako Pure Chemical Corporation, USA); anti-ApoE (Rabbit, Abcam, USA), anti-MAG (rabbit, Cell Signaling Technology, USA), anti-CNPase (rabbit, Cell Signaling Technology, USA), Anti-PU.1/SPI1 (Rabbit, Abcam, USA), Anti-C/EBPβ (Rabbit, Abcam, USA), Anti-ICSBP (IRF8) β (Mouse, Santa Cruz Biotechnology, USA), Anti-Smad2/3 (rabbit, Cell Signaling Technology, USA), p-STAT3 (Y705) (rabbit, Cell Signaling Technology, USA), STAT3 (rabbit, Cell Signaling Technology, USA), β-actin (rabbit, Cell Signaling Technology, USA), anti-GAPDH (rabbit, Cell Signaling Technology, USA), anti-β-tubulin (rabbit, Cell Signaling Technology, USA) overnight at 4 °C, followed by horseradish peroxidase (HRP)-linked secondary antibodies (Cell Signaling Technology, USA) for 1 h at room temperature. Pierce™ ECL Western Blotting Substrate was used to incubate PVDF membrane with transferred protein, then exposed with autoradiography film (HyBlot CL). Some PVDF membranes were reblotted after being treated with stripping buffer (Thermo Fisher Scientific, Waltham, MA, USA). Protein expression was analyzed with the software ImageJ and normalized to β-actin/GAPDH/β-tubulin expression. The original/full-blot images can be found in the supplemental file ‘sup WB original’.

### Immunofluorescence staining

The frozen slice was blocked by 10% goat serum (Sigma, USA) for 1 h at room temperature, then incubated with the primary antibody of anti-GFAP (chicken, Millipore, USA; rabbit, Dako, Japan), anti-VIM (chicken, Abcam, USA), anti-SerpinA3N (goat, R&D Systems), anti-Iba1 (rabbit, FUJIFILM Wako Pure Chemical Corporation, USA), anti-MBP (rabbit, Cell Signaling Technology, USA) at 4 °C overnight, washed three times, then incubated with the fluorescence-labeled second antibody (Invitrogen, USA) 1 h at room temperature; washed three times, and mounted with DAPI. Images were captured with a confocal laser-scanning microscope (Zeiss LSM710, USA) or Fluorescence Microscope BZ-X800 (KEYENCE, Japan).

### Electron microscopy

Mice were transcardially perfused with 0.1 M Millonigs buffer containing 4% paraformaldehyde and 2.5% glutaraldehyde; transversely-cut spinal cord samples at the levels of C3-C5 from the ventral column were dehydrated and embedded in PolyBed (PolySciences); ultrathin sectioned; stained with uranyl acetate and lead citrate, and imaged using a JEOL JEM 1400 Plus transmission electron microscope. Astrocytes and astrocytic processes were identified by the presence of the intermediate filament glial fibrillary acidic protein (GFAP) and glycogen clusters. GFAP presented as 10 nm filaments with characteristic bundling; glycogen granules presented as irregularly clustered puncta.

### Statistics

Data in figures were presented as mean ± SEM. All the statistical analyses for the gene transcript counts from Nanostring access were performed using the NanoString nSolver recommended test (heteroscedastic Welch’s t-Test). All other statistical analyses were performed using Prism (GraphPad). Statistical analyses to compare the mean values for multiple groups were performed by two-way ANOVA with Bonferroni post-hoc test for multiple comparisons. Comparisons of two groups were performed using a two-tailed unpaired t-Test. *p < 0.05, **p < 0.01, ***p < 0.001 and the *p*-value between 0.1 and 0.05 is directly shown above the columns or indicated as ^#^*p*.

## Results

### Adult-onset CNS myelin sulfatide deficiency induces mild cognitive impairment without disrupting oligodendrocyte/myelin homeostasis or cell death

**Fig. 1:**
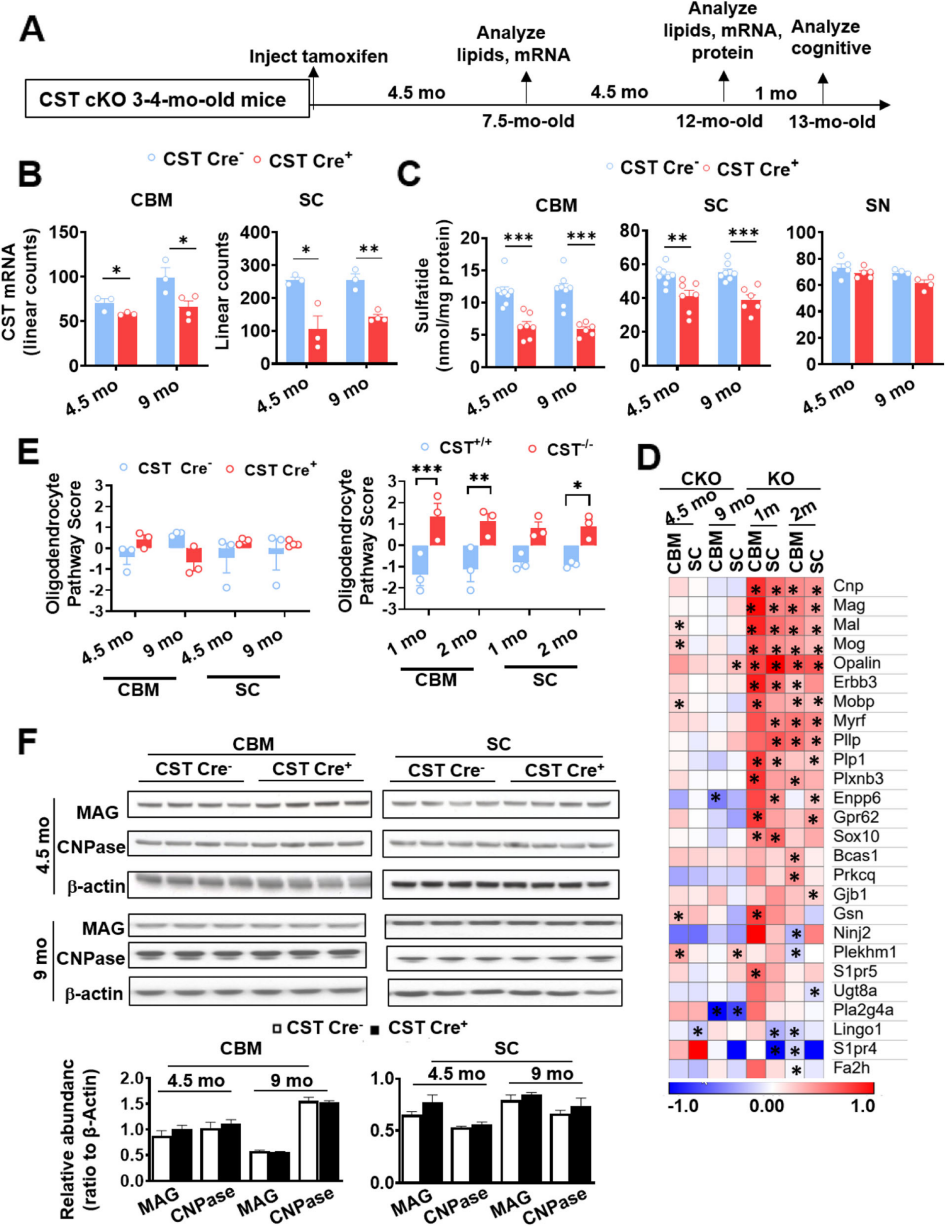
A novel inducible myelinating glia-specific CST cKO mouse model recapitulates AD-like adult-onset CNS-specific sulfatide loss without affecting oligodendrocyte/myelin homeostasis. **(A)** Tamoxifen injection and tissue harvest/analysis protocol. CST Cre^−^ and CST Cre^+^ mice were injected with tamoxifen at 3–4 mo of age and sacrificed at 4.5 and 9 mo post-injection, at 7.5 and 12 mo of age, respectively. (**B)** RNA extracted from the cerebrum (CRM) and spinal cord (SC) of CST Cre^−^ and CST Cre^+^ mice was assessed using the NanoString nCounter mouse Neuroinflammation Panel, linear mRNA counts for the *Cst* (*Gal3st1*) gene are shown. NanoString nSolver recommended test (heteroscedastic Welch’s t-Test), *n* = 3–4. **(C)** Lipid extracts from CRM, SC, and sciatic nerve (SN) of CST Cre^−^ and CST Cre^+^ mice were assessed by shotgun lipidomics; total sulfatide levels are shown as nmol/mg of total protein. See Fig. S2B-F for additional lipid classes. Two-way ANOVA with Bonferroni posthoc test for multiple comparisons, *n* = 4–8. (**D)** Heatmap displaying log2 fold changes of oligodendrocyte/myelin function-related genes included in the Nanotring panel (except *Gal3st1,* which is shown separately) from CRM, SC of CST cKO mice, as well as from 1 and 2 mo old CST KO mice for comparison, relative to their respective controls. Heteroscedastic Welch’s t-Test, n = 3–4. *P*-value is displayed within each heat map cell (*p < 0.05). (**E)** Oligodendrocyte pathway scores were obtained using nSolver 4.0 Advanced Analysis (NanoString Technologies). Two-way ANOVA with Bonferroni posthoc test for multiple comparisons, n = 3–4. **(F)** CRM and SC NP40 homogenate supernatants were assessed by Western blot using antibodies against myelin-associated glycoprotein (MAG) and 2′,3′-Cyclic nucleotide 3′-phosphodiesterase (CNP). Two-tailed unpaired t-Test, n = 3–4. *p < 0.05, **p < 0.01, ***p < 0.001. Data represent the mean ± S.E.M

Constitutive sulfatide depletion in CST knockout (CST^−/−^) mice results in abnormal myelin development and maintenance leading to tremors and ataxia [50, 55, 56]. Seeking to overcome the developmental consequences of germline genetic manipulation and to better model AD-like adult-onset brain sulfatide loss, we generated a CST floxP/floxP (CST^fl/fl^) mouse line (Fig. S1). To generate a novel tamoxifen-inducible myelinating glia-specific CST conditional knockout mouse (CST cKO) model, we crossed CST^fl/fl^ mice with the Plp1-CreERT mouse line, where CreERT is driven under the transcriptional control of the regulatory sequences of the myelin proteolipid protein (*Plp*) gene, which is abundantly expressed in oligodendrocytes and to a lower extent in Schwann cells [57]. CST^fl/fl^/Plp1-CreERT^+^ (CST Cre^+^) and CST^fl/fl^/Plp1-CreERT^−^ (CST Cre^−^) control mice were both treated with tamoxifen once they reached adulthood (i.e., 3–4 mo of age), a stage at which myelin has been largely developed [58], and assessed 4.5 and 9 mo post-injection (Fig. 1A). CST mRNA levels were several-fold higher in the spinal cord compared to the cerebrum and were significantly reduced in CST Cre^+^ mice compared to controls in both CNS regions at both time points analyzed (Fig. 1B). Our shotgun lipidomics studies revealed that CST Cre^+^ mice displayed a significant and progressive reduction of sulfatide content in both CNS regions compared to controls (Fig. 1C). Consistent with the much lower expression of Plp1 in Schwann cells, PNS (i.e., sciatic nerve) sulfatide levels were not significantly altered at either time point (Fig. 1C). CST^−/−^ mice, which are completely depleted of sulfatide, were used as a lipidomics negative control (Fig. S2A), demonstrating the specificity of our methodology. On the other hand, cerebroside, the precursor of sulfatide, was not significantly altered in CST Cre^+^ mice compared to controls (Fig. S2B). Similarly, total levels of sphingomyelin, another myelin-enriched sphingolipid, and ceramide, the central mediator of sphingolipid metabolism, were not significantly altered in the CNS of CST Cre^+^ mice (Fig. S2C,D). Other major phospholipid classes (e.g., phosphatidylcholine (PC) and phosphatidylserine (PS)) were also not significantly altered (Fig. S2E,F) in sulfatide deficient mice. To determine the effects of adult-onset CNS-specific sulfatide deficiency on oligodendrocyte/myelin homeostasis, we performed a comprehensive multiplex gene expression analysis using total RNA isolated from bulk tissue of two CNS regions (i.e., cerebrum and spinal cord) of CST cKO taking advantage of the Nanostring technology [59]. The results demonstrated that none of myelin-related genes were robustly altered in CST Cre^+^ mice compared to their respective controls (Fig. 1D). Consistently, oligodendrocyte function pathway scores were unaltered in CST Cre^+^ mice at every region/time point examined (Fig. 1E). Constitutive CST^−/−^ mice, which display mild losses of myelin-enriched lipids, dramatic losses of specific myelin proteins, and abnormal ultrastructural paranodal and internodal myelin [50, 55] were also assessed as a comparison at two different time points (1 and 2 mo). Conversely, multiple myelin-related genes were upregulated in CST^−/−^ mice in all CNS regions/time points analyzed (Fig. 1D). And oligodendrocyte function pathway scores were also significantly increased in CST^−/−^ mice in all the CNS regions/time points examined (Fig. 1E). We then assessed the levels of some major myelin proteins in CST cKO mice using bulk tissue homogenates from cerebrum and spinal cord, including myelin-associated glycoprotein (MAG), a myelin-specific protein that is restricted to the inner internodal layer and was previously shown to be dramatically reduced in adult CST^−/−^ mice [50] and cyclic nucleotide phosphodiesterase (CNP), a major non-compact myelin protein. None of the myelin-specific proteins assessed were significantly altered at any of the time points analyzed (4.5 and 9 mo post-induction) (Fig. 1F). Meanwhile, TUNNEL staining did not show obvious cell death in the brains of CST cKO or KO mice (Fig. S2G).

**Fig. 2:**
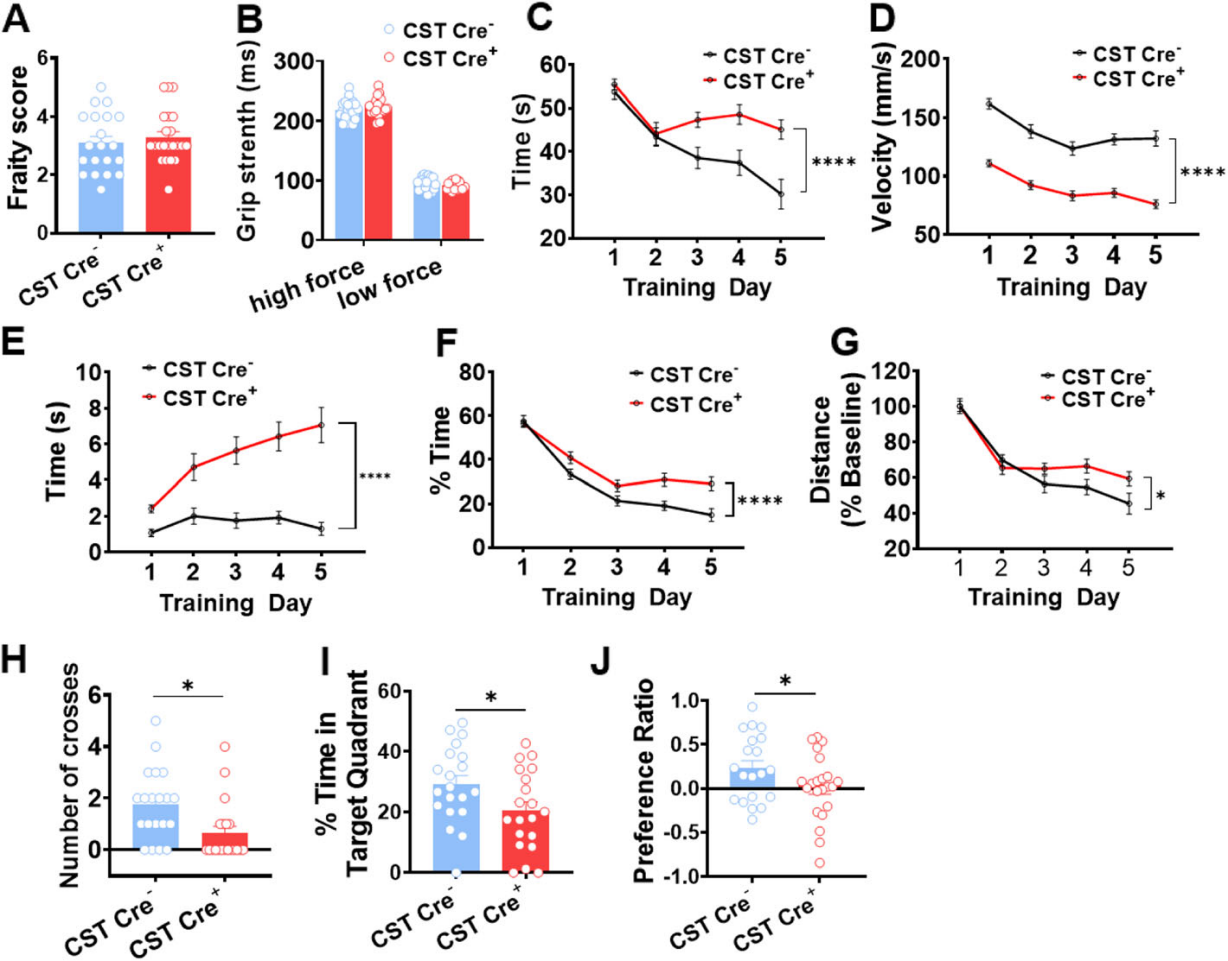
Adult-onset sulfatide deficiency caused cognitive impairment**.** The following behavior paradigms were run on CST cKO mice 10 mo post-injection. (**A)** Frailty Index. **(B)** Grip strength test. **(C-G)** Acquisition phase of the Morris Water Maze (MWM) test using a hidden platform, Two-way ANOVA *p*-value for the genotype effect in MWM test is shown between the data lines. **(C)** Swim time (latency). **(D)** Swim velocity. **(E)** Floating time (movement< 20 mm/s). **(F)** Thigmotaxis. **(G)** Swim distance that was normalized to the baseline of the first day of MWM test. (H-I) Probe trial of the MWM test on day 6, when platform was removed. **(H)** The times to cross the former platform location. **(I**) The percentage of time spent at the target quadrant. **(J)** Novel Object Recognition (NOR) test: Preference Ratio (time spent on novel object/time spent old object). (**A**, **H**-**J**) Two-tailed unpaired t-Test. *n* = 20–22. **(B-G)** Two-way ANOVA. n = 20–22. *p < 0.05, **p < 0.01, ***p < 0.001, ****p < 0.0001. Data represent the mean ± S.E.M

Behavioral studies were done to assess cognitive performance in CST cKO mice 10 mo after tamoxifen injection. We first assessed neuromotor function of CST cKO mice; no signs of major neuromotor impairment were found on CST Cre^+^ mice compared to CST Cre^−^ controls after performing a robust neurobehavioral screening using a battery of nine tests (described in Materials and Methods). Similarly, blinded frailty index scoring [48], including evaluation of the visual system and visual loss, did not show significant differences between CST Cre^−^ and CST Cre^+^ mice (Fig. 2A). Grip strength assessment revealed that CST Cre^+^ mice had normal muscle strength (Fig. 2B). We then assessed the potential effects of sulfatide deficiency on cognitive function using Morris water maze (MWM) and novel object recognition (NOR) paradigms. MWM studies revealed a dramatic genotype effect on average escape latency (time from start to goal) during spatial memory acquisition training, with CST Cre^−^ control mice displaying the expected progressively shorter latencies and CST Cre^+^ failing to do so, particularly on training days 3 to 5 (Fig. 2C). However, CST Cre^+^ mice also showed slower swim speeds (Fig. 2D) with increased floating time (movement < 20 mm/s) than controls (Fig. 2E). CST Cre^+^ mice also displayed a progressive increase of thigmotaxis (Fig. 2F), a commonly observed behavioral pattern that is thought to be related to anxiety/fear [60]. In an attempt to control for these swimming abnormalities that could cause confounding issues, we attempted to compare distance traveled to reach the platform by normalizing to day 1 (baseline) traveled distances. And even then, we observed a mild, but significant genotype effect during the initial learning phase (Fig. 2G). More importantly, during the memory consolidation phase recall-like task (probe trial) results revealed that CST Cre^+^ mice crossed the former platform location fewer times (Fig. 2H) and spent a significantly lower amount of time in the target quadrant compared to CST Cre^−^ controls (Fig. 2I). Notably, the latter measure is unlikely to be affected by the described differences in swim velocities/floating times/thigmotaxis. Notably, contrary to the weak and difficult to interpret genotype differences in learning abilities observed during the acquisition phase, the magnitude of the genotype differences on memory consolidation observed during the probe trial was pretty robust (Cre^+^ mice spent about one third less time in the target quadrant than controls), strongly favoring interpretation of these results as an impairment of cognitive abilities. Finally, non-spatial recognition memory, assessed by the NOR test, was also significantly impaired in CST Cre^+^ mice, which spent significantly less time exploring the novel object than CST Cre^−^ controls (Fig. 2J). It is important to note that the NOR paradigm should not be heavily influenced by potential mild locomotor defects. Taken together, MWM probel trial and NOR results led us to conclude that Cre^+^ mice display mild cognitive impairment. Intriguingly, this mild cognitive impairment was gender specific, with female CST cKO mice reaching significant differences, while male CST cKO mice did not even though they displayed borderline trends, demonstrated by the number of crosses in the probe test and the NOR test results (Fig. S2H).

Taken together, we provided multiple lines of evidence at the lipid, RNA, and protein levels, demonstrating that inducible myelinating glia-specific CST depletion leads to specific losses of CNS sulfatide with no major impact on overall oligodendrocyte/myelin homeostasis or cell death. Remarkably, our results demonstrated for the first time that adult-onset sulfatide deficiency, a specific event in AD, is sufficient to impair cognitive dysfunction, disrupting both spatial and non-spatial memory consolidation.

### CNS myelin sulfatide depletion induces a chronic immune/inflammatory response characterized by microgliosis and astrogliosis without infiltration of peripheral immune cells

**Fig. 3:**
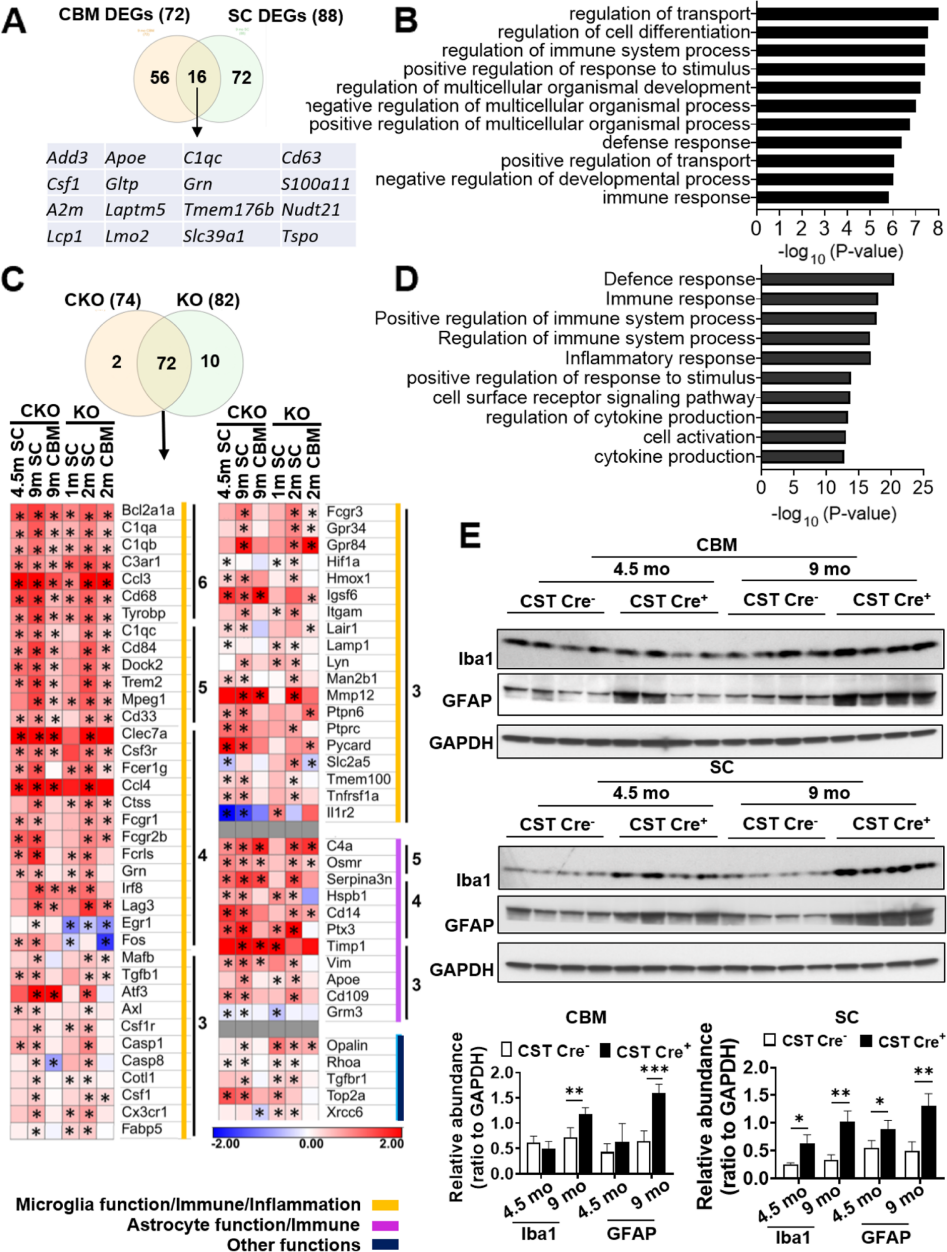
CNS sulfatide depletion induces a chronic immune/inflammatory response characterized by strong progressive activation of microglia and astrocytes. **(A)** Venn diagram showing the number of specific and shared differentially expressed genes (DEGs) in the CRM and SC of CST cKO mice compared to their respective controls from the Nanostring AD panel. Shared DEGs are listed. **(B)** Gene Ontology (GO) analysis for the 16 shared DEGs was performed using the Database for Annotation, Visualization and Integrated Discovery (DAVID). Top functions are listed**. (C)** Venn diagram showing the number of DEGs found in at least three groups from six groups of different CNS regions/time points from both CST cKO and CST KO mice. Heatmap displaying log_2_ fold changes of the 72 DEGs that were shared by both CST cKO and CST KO mice. DEGs were grouped into three major categories by lines of different color and ordered based on how many times they were significantly altered in six groups. See Fig. S3F for the list of the DEGs that were specific to CST cKO or CST KO mice. Heteroscedastic Welch’s t-Test, n = 3–4. *p*-values are displayed within each heat map cell (*p < 0.05). **(D)** List of the top functions from GO analysis for the 72 shared DEGs. **(E))** Western blot analysis from cerebrum and spinal cord of 4.5 mo and 9 mo post-injection CST cKO mice using antibodies against Iba1 and GFAP. Relative expression was quantified and plotted as a ratio to GAPDH**.** Two-tailed unpaired t-Test, *n* = 4. *p < 0.05, **p < 0.01. Data represent the mean ± S.E.M

Next, we were interested in unraveling the potential changes in the CNS related to sulfatide loss, particularly in regards to AD, given the mild cognitive impairment observed in sulfatide deficient mice and the sulfatide loss in AD etiology [31, 61]. We used the Nanostring mouse AD panel, which consists of 770 genes representing different pathways related to AD to assess CST cKO mice (9 mo post-injection) in two CNS regions (i.e., cerebrum and spinal cord). The AD panel results revealed 16 overlapped differentially expressed genes (DEGs) in the cerebrum and spinal cord regions (Fig. 3A). Gene Ontology (GO) analysis was performed by inputting the 16 shared DEGs into the Database of Annotation, Visualization and Integrated Discovery (DAVID, https://david.ncifcrf.gov/), revealing that the top significantly altered biological themes included transport, cell differentiation/development and immune response (Fig. 3B).

To further address the immune response caused by sulfatide loss in detail, we used the Nanostring nCounter mouse neuroinflammation panel, which consists of 770 genes that represent 22 different pathways, primarily related to immune response/inflammation. We analyzed cerebrum and spinal cord from CST cKO mice at two time points (4.5- and 9-mo post-tamoxifen injection). Principal component analysis (PCA) showed that CST Cre^+^ groups could be separated from the corresponding CST Cre^−^ control samples (Fig. S3B). In addition, as expected, cerebral RNA profiles were different than spinal cord ones (Fig. S3A). 76 DEGs appeared in at least two of four groups (Fig. S3C). The vast majority (57 out of 76) of DEGs are involved in microglial/immune response function, and most of the remaining ones (10 out of 76) are engaged in astrocytic function, with three DEGs involved in matrix remodeling (Fig. S3C). GO analysis from the listed 76 DEGs demonstrated the top related functions were all involved in/related to immune responses (Fig. S3D).

We also performed the analysis of Nanostring mouse neuroinflammation panel on cerebrum and spinal cord of CST KO mice at 1 and 2 mo, whose sulfatide was completely depleted from the beginning of myelin development instead of adult-onset. Cerebrum of earliest time point of each model, which had the least changes, were excluded from the following overlapping analysis of DEGs to make the description more refined. 16 DEGs were shared between the three CST cKO groups, all of them related to microglia/immunity/inflammation and astrocyte function (Fig. S3E); while 24 DEGs were shared between the three altered CST KO conditions, all of them related to either microglia/immunity/inflammation or oligodendrocyte function (Fig. S3E). A total 84 DEGs were significantly altered in at least three of the six groups (including both CST cKO and CST KO), most of these DEGs (72 out of 84) were found in both CST cKO and CST KO mice (Fig. 3C). On the other hand, ten DEGs were unique to CST KO, all of them oligodendrocyte-related, while only two of them were unique to CST cKO (Fig. S3F). Similar to the analysis result from only CST cKO mice (Fig. S3C), the vast majority of the DEGs (56 out of 72, Fig. 3C orange line) found in both CST Cre^+^ and CST^−/−^ are involved in microglial function, and most of the remaining ones (11 out of 72, Fig. 3C purple line) are involved in astrocytic function. The top biological processes from GO analysis were also all immune responses (Fig. 3D). Consistently, pathway scores of astrocyte and microglia function, innate immune response, and inflammatory signaling were all increased in CST Cre^+^ mice compared to CST Cre^−^ controls (Fig. S4A), as well as in CST^−/−^ mice compared to respective CST^+/+^ controls (Fig. S4B); and epigenetic regulation pathway score was as the negative control (Fig. S4C). The microglia and astrocyte pathway score was upregulated in an age-dependent manner in both CNS regions (Fig. S4A, B). And western blot results with microglia and astrocyte activation marker (Iba1 and GFAP, respectively) also showed their progressive activation in cerebrum and spinal cord of CST cKO (Fig. 3E). The age-dependent activation of the astrocyte (used marker:*Gfap*) and microglia (used marker: *Cd68*, *Iba1* and *Cd11b*) were further confirmed by real-time qPCR results using CST KO brain of different ages (1 mo-, 2mo, 3 mo and 6 mo old) (Fig. S5A). These results suggested sulfatide deficiency induced similar microgliosis and astrogliosis, regardless of whether oligodendrocyte/myelin homeostasis was altered or not. Furthermore, Western blot results from GFAP and Iba1 also demonstrated that CNS of female CST cKO mice showed more serious activation of both astrocyte and microglia than that of male CST cKO mice compared to their respective controls (Fig. S6A).

Meanwhile, B-cell, dendritic cell, mast cell, T-cell, CD8 T-cell, Th1 cell or Treg-related genes were not altered in any of the groups examined (Fig. S5B, C), which implied no infiltration of peripheral immune cells into the CNS during the immune and inflammatory response after sulfatide deficiency.

### CNS myelin sulfatide deficiency induced AD-like neuroinflammation gene expression profiles, including disease-associated microglia and disease-associated astrocytes signatures

**Fig. 4:**
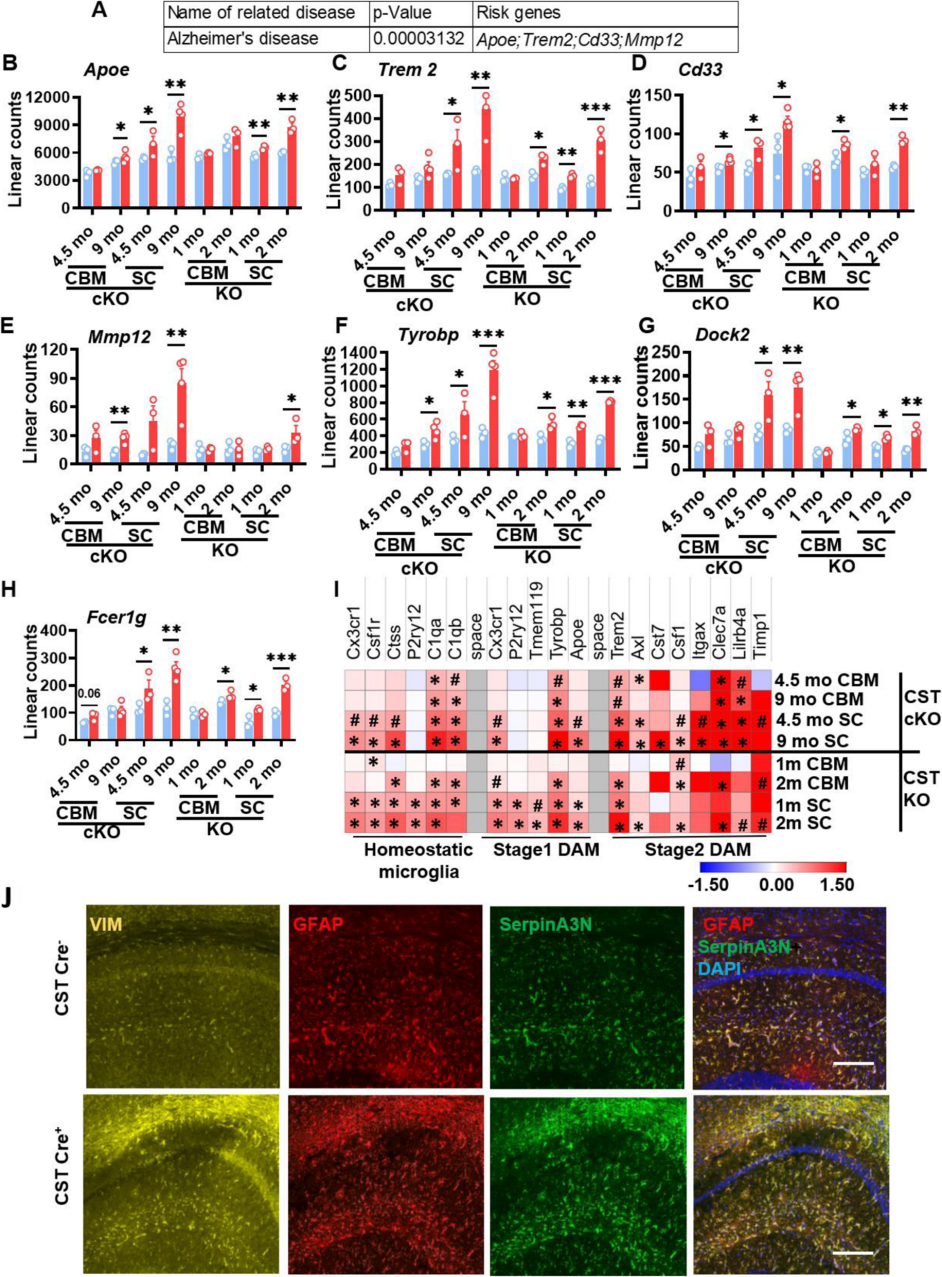
Sulfatide deficiency caused AD-like neuroinflammation, leading to disease-associated microglia and astrocytes signatures. **(A)** Gene list enrichment analysis using Enrichr for the 72 DEGs shared by both CST cKO and CST KO CNS (listed in Fig. 3C), The top 1 disease reaching a significant difference was Alzheimer’s disease with four AD risk genes: *Apoe*, *Trem2*, *Cd33*, and *Mmp12* among the DEGs. **(B-E)** Linear counts of each of these AD risk genes at different CNS regions/time points for CST cKO and CST KO, compared to their respective controls. (**F-H)** Three genes previously described as key causal regulators of immune networks for late-onset AD were also among the shared DEGs: *Tyrobp, Dock*, *Fcerg1*. **(I)** Heatmap displaying log_2_ fold changes of the homeostatic microglia and stage1/2 reactive microglia-specific genes (those included in the Nanostring panel) in the CNS of CST cKO and KO, compared to their respective controls. 0.05 < #*p* < 0.1, *p < 0.05. **(J)** Immunofluorescence staining on the hippocampus (CA1 region shown) of CST Cre^−^ and CST Cre^+^ brain 9 mo post injection using antibodies against typical markers of disease-associated astrocytes: VIM (yellow), GFAP (red) and SerpinA3N (green). Scale bar: 200 μm. **(B-I)** Heteroscedastic Welch’s t-Test, n = 3–4. *p < 0.05, **p < 0.01, ***p < 0.001. Data represent the mean ± S.E.M

To further analyze the resemblance of gene expression profiles with sulfatide deficiency, we first did gene enrichment analysis by inputting 72 DEGs from both CST cKO and KO CNS (Fig. 3C) into GWAS Catalog 2019 screening on Enrichr [62], which revealed that the top related disease was AD (Fig. 4A), with four AD risk genes being significantly upregulated under several conditions of sulfatide deficiency: *Apoe*, *Trem2*, *Cd33*, and *Mmp12* (Fig. 4A-E). Similarly, the top three genes previously described as causal regulators of late-onset AD (i.e., *Tyrobp*, *Dock*, *Fcerg1*) [11] were also significantly upregulated in the CNS of both CST cKO and KO (Fig. 4F-H). KEGG pathway enrichment analysis of the 72 overlapping genes revealed that several of the top pathways (e.g., cytokines, complement and Fc pathways) (Fig. S6B) were strongly linked to AD, with integrative network-based analysis having classified them as functional immune pathways for late-onset AD [11]. Both adult-onset or constitutive myelin sulfatide deficiencies led to early and strong activation of cytokine (e.g., *Ccl3, Ccl4, Csf3r, Osmr*) and complement pathway-related genes (e.g., *C1qC, C1qa, C1qb, C3, C3ar1*); similarly, the Fc pathway was also heavily activated (e.g., *Fcgr1, Fcgr2b, Dock*) (Fig. S6B).

Furthermore, single-cell transcriptomics has revealed specific microglial and astrocytic subtypes associated with AD, known as disease-associated microglia (DAMs) [15] and disease-associated astrocytes (DAAs) [16] in AD mice. First, multiple genes associated with homeostatic, stage 1 and 2 DAMs included in the Nanostring neuroinflammation panel were upregulated significantly in multiple sulfatide deficient conditions (Fig. 4I). Among these genes, *Apoe, Trem2,* and *Tyrobp* have been consistently linked with AD-specific microglia in both animal and human studies [13, 15, 63], and they were significantly upregulated in most myelin sulfatide deficient conditions. Meanwhile, GFAP, VIM, and SerpinA3N are DAA markers [16]. The level of mRNA tested by real-time qPCR (GFAP, Fig. S5A) or Nanostring (*Vim* and *Serpin*a3n, Fig. 3C) showed the increased expression of these DAAs. Consistently, the co-staining of these three antibodies confirmed that the reactive astrocytes in the CNS of sulfatide deficient mice were DAAs (Fig. 4J).

### CNS myelin sulfatide deficiency leads to marked astrogliosis and microgliosis within myelin-enriched brain regions

**Fig. 5:**
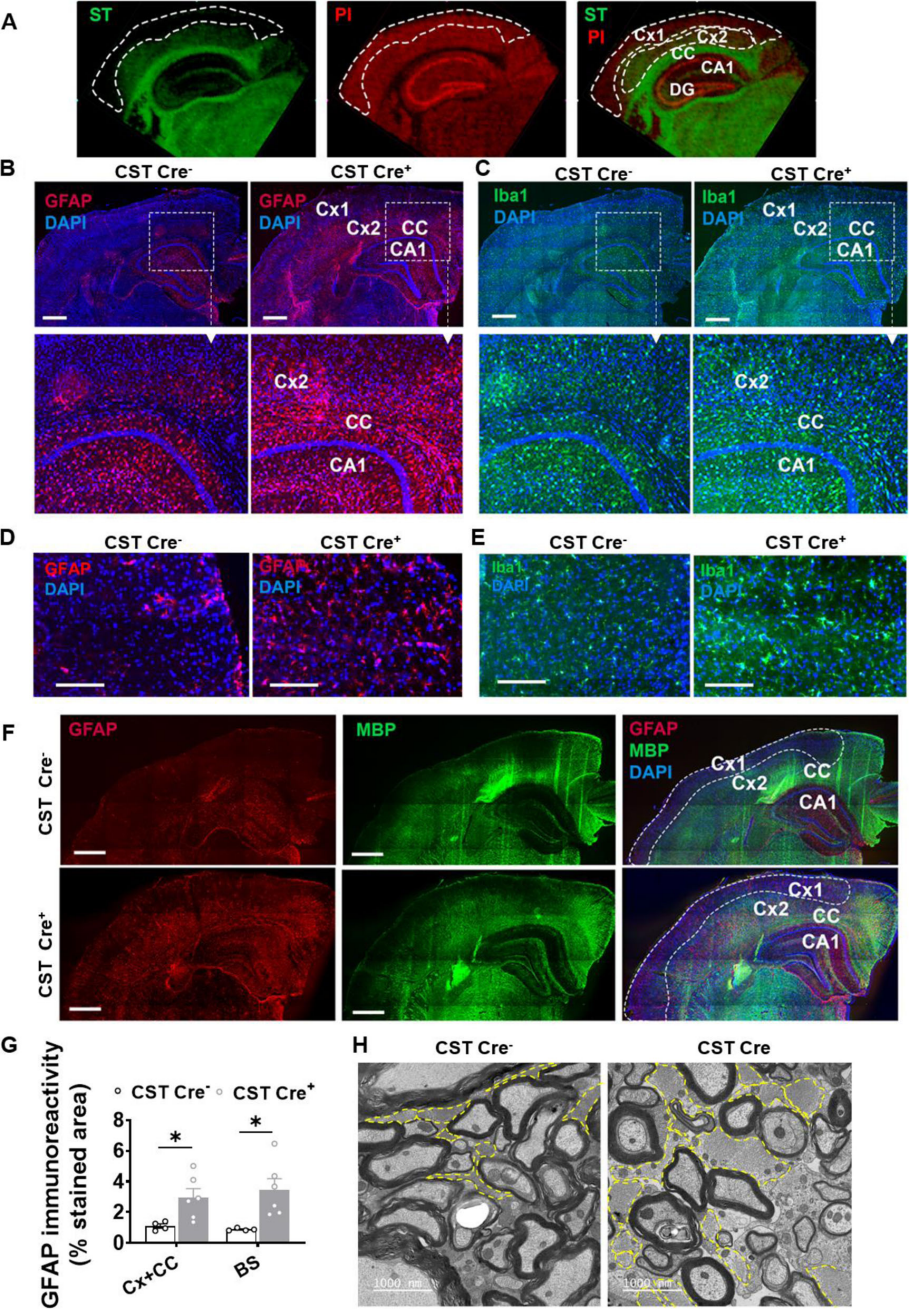
Adult-onset myelin sulfatide deficiency lead to marked astrogliosis and microgliosis within specific myelin-enriched brain regions. **(A)** MALDI-imaging of an adult WT mouse brain displaying the distribution of the most abundant sulfatide species (ST(N24:1), *m/z* 888, green), as well as a neuronal rich phosphatidylinositol species (PI(38:5), *m/z* 883.5, red). **(B-E)** Representative immunofluorescence images of CST cKO Cre^−^ and CST cKO Cre^+^ mice 9 mo post-injection using antibodies against GFAP (red) and Iba1 (green) on brain (**B,C**) and brain stem (**D,E**). Scale bar: 500 μm (**B**, **C**) and 200 μm (**D,E**). Quantification of GFAP IF staining area percentage on (cortex + corpus callosum) and brain stem was shown in (**G**). **(F)** Co-staining of GFAP (red) and myelin basic protein (MBP, green) in brain of CST cKO Cre^−^ and CST cKO Cre^+^ mice 9 mo post-injection. Scale bar: 500 μm. (**G**) GFAP relative expression on (cortex + corpus callosum) and brain stem was quantified and plotted as a IF staining area percentage**. (H)** Electron microscopy images from the spinal cord of CST cKO Cre^−^ and CST cKO Cre^+^ mice 11 mo post-injection. Astrocytes, identified by the intermediate filament GFAP and glycogen clusters were highlighted by the yellow dash line. Cx: cortex (Cx1: outer cortex without enriched sulfatide; Cx2: inner cortex with enriched sulfatide); CC: corpus callosum; CA1: Cornu Ammonis 1 region of the hippocampus; DG: hippocampal dentate gyrus

Our above results in both CST cKO and KO CNS demonstrated that the onset/magnitude of the inflammatory responses tended to occur earlier/more extensively in the spinal cord than in the brain of genetically modified sulfatide deficient mice. Although in relative terms sulfatide losses in CST cKO mice were more dramatic in the cerebrum compared to the spinal cord, in absolute terms, the amount of sulfatide lost in spinal cord was greater than that in cerebrum (Fig. 1C), which suggested that microglia/astrocyte activation and the related immune/inflammation response after myelin sulfatide deficiency might be connected with the extent of sulfatide deficiency/distribution and microglia/astrocyte distribution in the CNS. We then assessed the spatial association between sulfatide, astrogliosis and microgliosis in both CST cKO and CST KO mice to clarify the related mechanism further. First, our matrix-assisted laser desorption/ionization mass spectrometry (MALDI-MS) imaging results displayed the typical distribution of sulfatide in the brain, demonstrating that although sulfatide is most abundant within white matter-rich regions like the corpus callosum (CC) and the fimbria, it is also present in the inner cortical layers (V-VI), the stratum lacunosum moleculare of the hippocampus, and the thalamus (Fig. 5A). Immunostaining of GFAP or Iba1 on cerebrum revealed a dramatic upregulation of GFAP and Iba1 in brain regions where sulfatide is present/enriched, including CC and AD relevant brain regions, e.g., the inner cortical layers and partial hippocampus substructure in both CST cKO (Fig. 5B, C) and CST KO mice (Fig. S7A,B). Western blot results using GFAP antibody on cerebral substructures, i. e., cortex and hippocampus, of CST KO mice reconfirmed the above observation (Fig. S7E). Brain stem, a myelin-rich brain region, also showed abundant activation of microglia and astrocyte in both CST cKO (Fig. 5D, E) and CST KO mice (Fig. S7C,D). Co-staining of the myelin marker myelin basic protein (MBP) and GFAP in the cerebrum further confirmed the association between sulfatide/myelin enriched regions and astrocyte activation in both CST cKO (Fig. 5F) and CST KO mice (Fig. S7G). Furthermore, electron microscope images from the spinal cord of CST cKO mice after 11 mo post tamoxifen injection revealed numerous hypertrophic astrocytic processes surrounding myelin in CST cKO mice (Fig. 5H), suggesting that the astrocytes might sense the lack of sulfatide.

The reactive astrocytes and microglia in the brains of sulfatide-deficient mice were obviously hypertrophic (Fig. S8A). To clarify whether they were proliferative, we analyzed their cell-specific gene expression profile. Results pointed to activation of CNS resident microglia, without obvious microglial proliferation, as *Cd68* (in macrophages and activated microglia) (Fig. S8B) and a lot of the microglial function-related genes (e.g., *Cd84, Trem2*, *Tyrobp*, *Dock2*, and *Mprg1*) were upregulated significantly (Fig. 3C), while some resting microglia/macrophage markers (e.g., *Cd163* and *Trem119*) were not significantly altered (Fig. S8B). Similarly, astrocytes also seemed to be activated, but not proliferative as reactive astrocyte genes were significantly upregulated (e.g., *Vim*, *Osmr,* and *Serpina3n*), while resting astrocyte genes (e.g., *Itga7* and *S100b*) were not significantly altered (Fig. 3C; Fig. S8C).

### Myelin sulfatide deficiency led to ApoE upregulation, but sulfatide deficiency induces neuroinflammation independently of ApoE

**Fig. 6:**
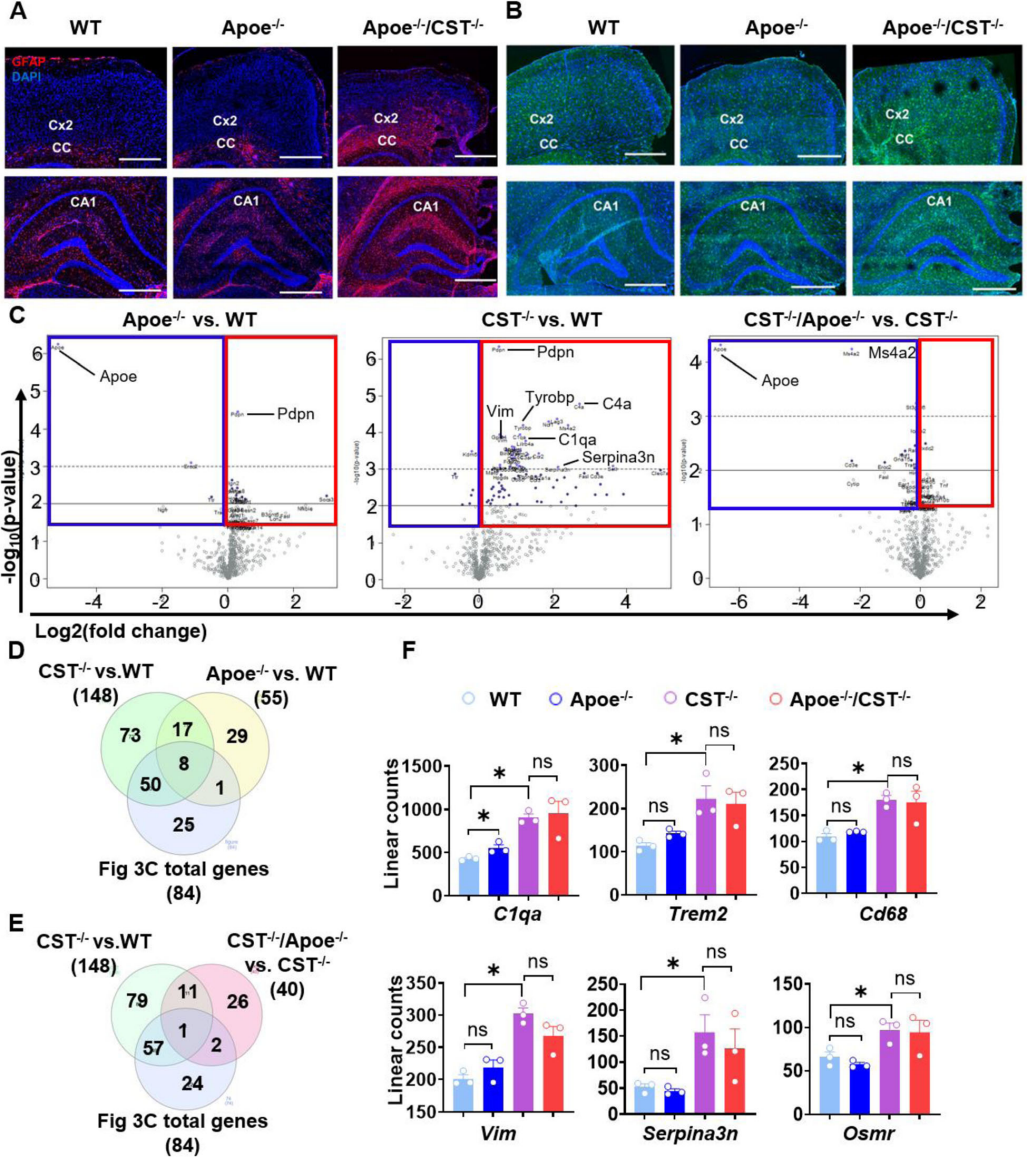
Myelin sulfatide deficiency induced AD-like neuroinflammation even in the absence of ApoE. **(A,B)** Representative immunofluorescence images from brain of 3-mo-old ApoE^+/+^/CST^+/+^, ApoE^−/−^/CST^+/+^, and ApoE^−/−^/CST^−/−^ mice using antibodies against GFAP (red) and Iba1 (green). Cx: cortex; CC: corpus callosum; CA1: Cornu Ammonis 1 region of the hippocampus. Scale bar: 200 μm. **(C-F)** The brain mRNA from the four genotypes (ApoE^+/+^/CST^+/+^, ApoE^−/−^/CST^+/+^, ApoE^+/+^/CST^−/−^and ApoE^−/−^/CST^−/−^) was accessed using NanoString neuroinflammation panel. **(C)** Volcano plot displaying -log_10_ p-value and log_2_ fold change for each gene to show the CST KO effect (middle) and the ApoE KO effect in the presence (CST^+/+^, left) or absence of sulfatide (CST^−/−^, right). **(D)** Venn diagrams showing the number of specific and shared upregulated DEGs from ApoE^−/−^ vs. WT, CST^−/−^ vs. WT and the DEGs listed in Fig. 3C. The gene lists were shown in Fig. S9. **(E)** Venn diagrams showing the number of specific and shared upregulated DEGs from CST^−/−^ vs.WT, ApoE^−/−^/CST^−/−^ vs. CST^−/−^ and the DEGs listed in Fig. 3C. The gene lists were shown in Fig. S9. **(F)** Linear counts of several typical microgliosis- and astrogliosis-related genes in ApoE^+/+^/CST^+/+^, ApoE^−/−^/CST^+/+^, ApoE^+/+^/CST^−/−^and ApoE^−/−^/CST^−/−^ mouse brain. Heteroscedastic Welch’s t-Test, *n* = 3. *p < 0.05, ns: no statistical significance

ApoE is mainly produced by astrocytes and is the major extracellular lipid carrier in the CNS, transporting multiple lipids, including sulfatide. It is important to note that, in the context of AD, ApoE is necessary to bring down brain sulfatide levels, as ApoE transports brain sulfatide and modulates its turnover [23]. We found that ApoE was upregulated in the CNS of CST cKO and KO (Fig. 4B). Therefore, our results showed that myelin sulfatide deficiency and ApoE upregulation formed feedback. Moreover, previous studies revealed a convergent ApoE pathway from aging, amyloid, and tau [64]. We thus tried to clarify whether ApoE is involved in sulfatide deficiency-induced neuroinflammation using ApoE and CST double KO mice (ApoE^−/−^/CST^−/−^). Our GFAP and Iba1 immunofluorescence showed that ApoE^−/−^ mice alone did not display any obvious astrocyte or microglial activation (Fig. 6A, B, middle panel). Moreover, we observed the spatiotemporal distribution and intensity of strong astrogliosis and microgliosis in the brains of 3-mo-old ApoE^−/−^/CST^−/−^ mice (Fig. 6A, B, right panel) fully resembled that observed in CST^−/−^ mice (Fig. S7A,B). Volcano plots of mRNA profiling result from cerebrum using the Nanostring neuroinflammation panel clearly showed ApoE KO did not result in a similar DEG profile to that of CST^−/−^ cerebrum (Fig. 6C, left and middle panel); and ApoE absence had a limited effect on the neuroinflammation-related DEGs observed in CST KO through comparing ApoE^−/−^/CST^−/−^ to CST^−/−^ (Fig. 6C, right panel). Specifically, CST^−/−^ brain had much more DEGs (148 DEGs) than ApoE^−/−^ brain (55 DEGs) (Fig. 6D). 50 DEGs of the 84 DEGs listed in Fig. 3C shared only with CST^−/−^ but not ApoE^−/−^, and only eight DEGs of 84 DEGs listed in Fig. 3C were shared with both CST^−/−^ and ApoE^−/−^ (Fig. 6D, Fig. S9A). Consistently, ApoE absence had little impact on the DAMs and DAAs profile caused by CST KO because only three DEGs from 84 DEGs listed in Fig. 3C were shared with the DEGs from ApoE^−/−^/CST^−/−^ vs. CST^−/−^ (Fig. 6E, Fig. S9B). For example, the typical microgliosis-related genes (*Trem2, Cd68*) and astrogliosis-related genes (*Vim, Serpina3n, Osmr*) were not altered by ApoE KO in either ApoE^−/−^ brain compared to WT or ApoE^−/−^/CST^−/−^ brain compared to CST^−/−^ (Fig. 6F). Even though the complement factor *C1qa* were upregulated in both ApoE^−/−^ and CST^−/−^ brain, *C1qa* level was much higher in CST^−/−^ brain than in ApoE^−/−^ brain, and no differences were observed between CST^−/−^ and ApoE^−/−^/CST^−/−^ brain (Fig. 6E). Therefore, our results showed that myelin sulfatide deficiency and ApoE upregulation formed feedback, but myelin sulfatide deficiency could subsequently activate DAMs and DAAs independently of ApoE because once sulfatide levels are decreased, in this case via genetic manipulation, apoE is not required for activation of DAMs and DAAs.

### CNS myelin sulfatide deficiency induces astrogliosis and ApoE upregulation independently of microgliosis

**Fig. 7:**
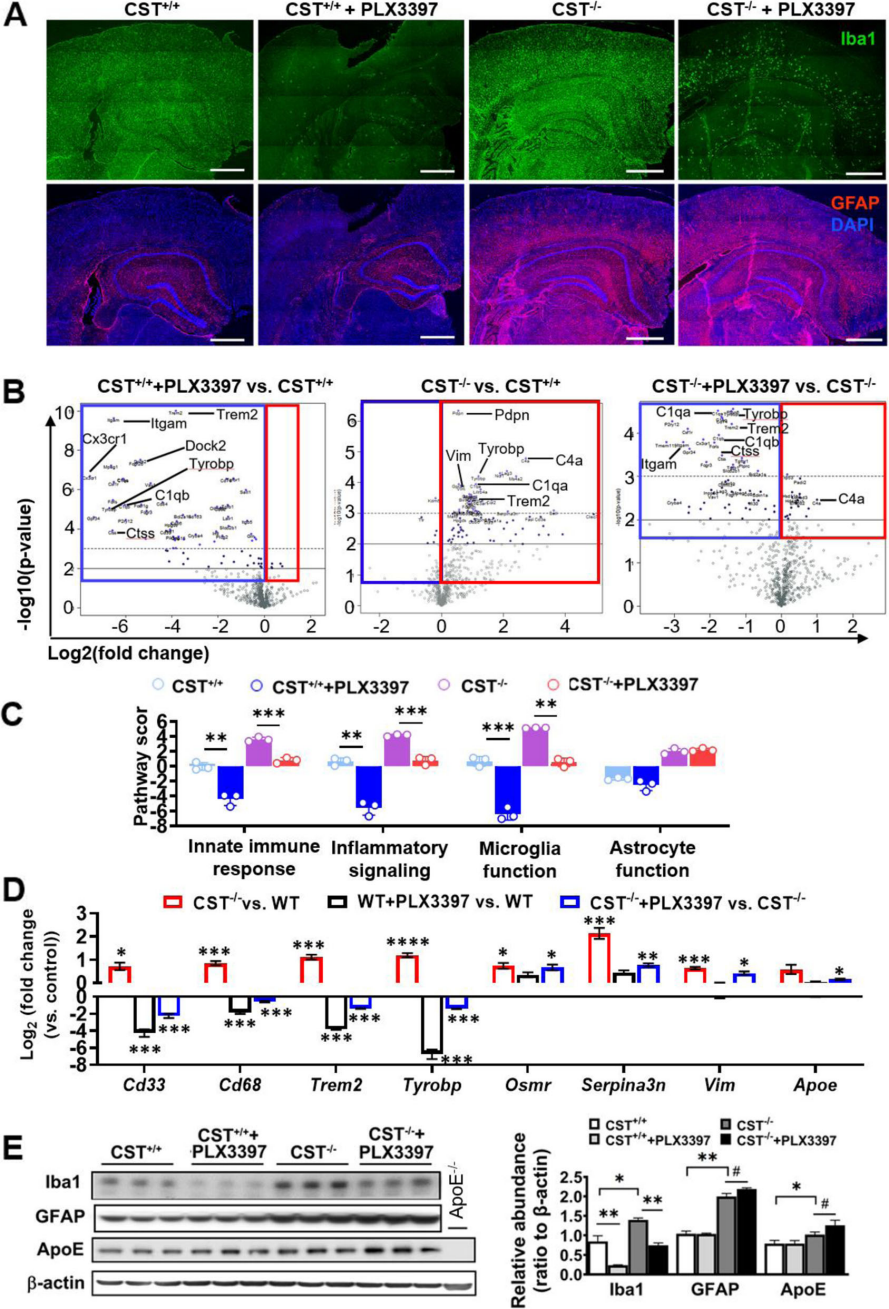
Sulfatide deficiency-induced astrogliosis and upregulated ApoE were not secondary to microgliosis. CST^+/+^ and CST^−/−^ mice were fed PLX3397-containing or control chow-like diet from 1 to 3 mo of age. **(A)** Representative immunofluorescence images from brain sections using antibodies against Iba-1 (green) and GFAP (red); nuclei were stained with DAPI; See Fig. S10A for higher magnification and merged images. Scale bar: 500 μm. **(B-D)** Brain mRNA from four groups (CST^+/+^, CST^+/+^+PLX3397, CST^−/−^, and CST^−/−^ + PLX3397 mice) was accessed using NanoString neuroinflammation panel. (**B)** Volcano plot displaying -log_10_ p-value and log_2_ fold change for each gene to show the CST KO effect (middle) and drug-effect within CST^+/+^ (left) and CST^−/−^ (right) brain. See Fig. S10B-D for PCA and Venn diagrams showing the number of specific and shared DEGs between treatments. **(C)** NanoString pathway score analysis for astrocyte function, microglia function, innate immune response, and inflammatory signaling for each of the four groups. Two-way ANOVA with Bonferroni post-hoc test for multiple comparisons, n = 3. **(D)** Fold changes of several typical microgliosis or astrogliosis related genes in CST^+/+^ and CST^−/−^ brain with or without PLX3397 treatment to display the drug effects on microglia and astrocytes. Heteroscedastic Welch’s t-Test, n = 3. **(E)** Western blot analysis from cerebrum homogenates using antibodies against Iba-1, GFAP, ApoE and β-actin. Two-tailed unpaired t-Test, n = 3. ^#^p < 0.1, *p < 0.05, **p < 0.01, ***p < 0.001. Data represent the mean ± S.E.M

Although we confirmed sulfatide deficiency resulted in microgliosis and astrogliosis in an ApoE independent manner, we also found that ApoE was upregulated in CST cKO and KO CNS subsequently to microglia/astrocyte activation. To further study the related mechanism of ApoE upregulation and microglia/astrocyte activation under sulfatide deficient conditions, we used PLX3397, an inhibitor of the colony stimulating factor 1 receptor (CSF1R) to eliminate microglia brain-wide [65]. Consistent with previous reports [65, 66], PLX3397 treatment eliminated the vast majority of microglia in CST^+/+^ brains and robustly eliminated a large portion of microglia in CST^−/−^ brains (Fig. 7A; Fig. S10A). Unexpectedly, substantial microglial elimination had absolutely no impact on astrogliosis (Fig. 7A; Fig. S10A). PCA from NanoString result showed the RNA expression profiles from cerebrum of 3-mo-old CST^+/+^, CST^+/+^ + PLX3397, CST^−/−^, and CST^−/−^ + PLX3397 mice could be separated well (Fig. S10B). Volcano plots of the RNA access clearly showed how PLX3397 treatment, as expected, led to a dramatic downregulation of genes that are predominantly or exclusively expressed by microglia in both CST^+/+^ and CST^−/−^ mice (Fig. 7B). Pathway score analysis also showed the PLX3397 treatment downregulated immune/inflammation responses and microglial function both in WT and CST KO mouse brain, but on the contrary, not astrocyte function (Fig. 7C). 34 of the 72 DEGs described in Fig. 3C that showed to be upregulated under sulfatide deficient conditions were the microglia-enriched genes and were significantly “knocked down” by PLX3397 (Fig. S10C). Among them were the AD-related microgliosis genes *CD33, Cd68, Trem2*, and *Tyrobp* (Fig. 7D). However, astrocyte-related genes (e.g., *Vim*, *Osmr, Serpina3n*) were significantly upregulated in both CST^−/−^ mice with or without PLX3397 treatment (Fig. 7D; Fig. S10D). Western blot results of GFAP and Iba1 were consistent with the mRNA results (Fig. 7E). Surprisingly, ApoE mRNA and protein levels were both increased in CST KO brain after microglia depletion by PLX3397 (Fig. 7D, E), which suggested that the increased ApoE in the CNS after sulfatide deficiency mainly came from reactive astrocytes. Meanwhile, the slight ApoE upregulation caused by microglia elimination might indicate the compensatory ApoE producing in astrocytes after microglia depletion. Taken together, our results demonstrated that sulfatide deficiency-induced astrogliosis and ApoE upregulation was not secondary to, but rather independent of microgliosis.

### Sulfatide deficiency-induced astrogliosis and microgliosis seem to be driven by activation of STAT3 and PU.1/ SPI1 transcription factors, respectively

**Fig. 8:**
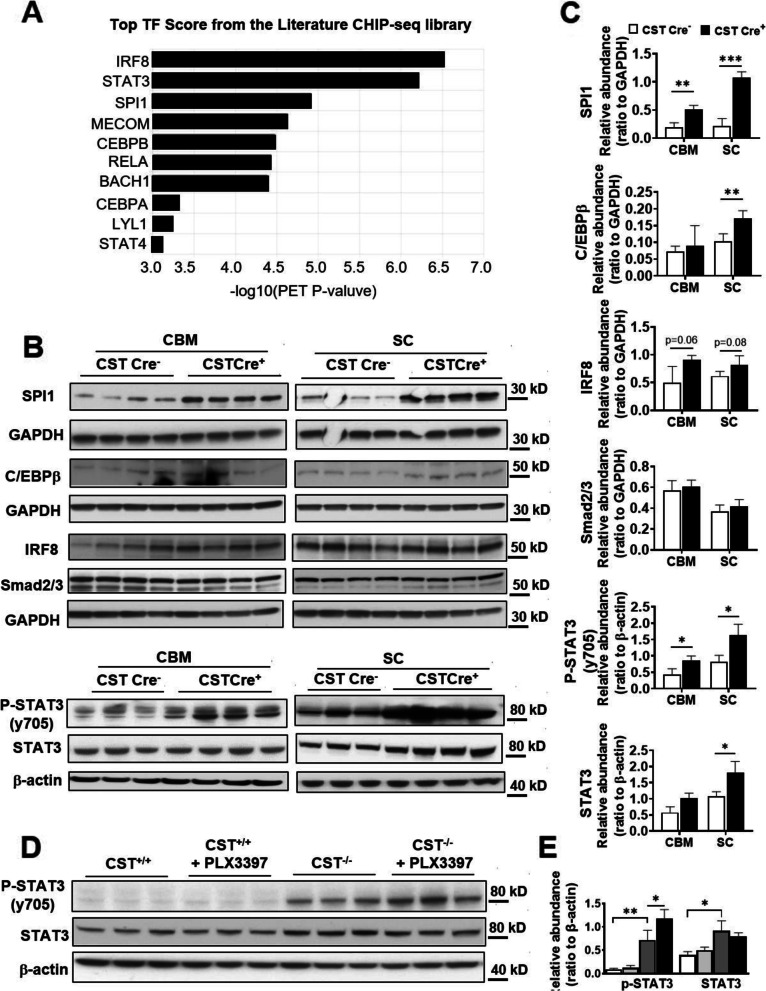
Myelin sulfatide deficiency resulted in upregulation of SPI1, STAT3, and C/EBPβ transcription factors in CNS. **(A)** The transcription factors (TF) score by using ChEA3 (ChIP-X Enrichment Analysis Version) with 72 DEGs listed in Fig. 3C. **(B)** Western blot analysis from cerebrum and spinal cord of 9 mo post-injection CST cKO mice using antibodies against transcription factors SPI1, C/EBPβ, IRF8, Smad2/3, STAT3 and its Y705 phosphorylation. **(C)** Relative expression was quantified and plotted as a ratio to GAPDH or β-actin for **(B). (D)** Western blot analysis from cerebrum of CST^+/+^ and CST^−/−^ mice with or without PLX3397 treatment using antibodies against STAT3 and its Y705 phosphorylation and β-actin. **(E)** Relative expression was quantified and plotted as a ratio to β-actin for **(D)**. **(C,E)** Two-tailed unpaired t-Test, n = 3. *p < 0.05, **p < 0.01. Data represent the mean ± S.E.M

To further study the molecular mechanism underlying sulfatide deficiency-induced neuroinflammation, we analyzed transcription factors (TF) scores using ChEA3 (ChIP-X Enrichment Analysis Version 3). The top targets included IRF8, STAT3, SPI1, and C/EBPβ (Fig. 8A), which have been reported to be involved in the activation of microglia or astrocytes [17, 18, 20, 67], and SPI1 is one of AD risk and microglia-enriched genes [20]. We used WB with cerebrum and spinal cord of 9 mo post-injection to confirm these software-based predictions. Strikingly, SPI1 was dramatically upregulated at the protein level (Fig. 8B, C), although Irf8 showed slightly upregulated in spinal cord of CST cKO (Fig. 8B,C). C/EBPβ was reported to play roles in neuroinflammation and is the mediator of ApoE4 expression in AD, and its protein expression level showed significant upregulation in spinal cord (Fig. 8B, C). Meanwhile, STAT3 was also dramatically upregulated at both phosphorylation and protein expression levels (Fig. 8B, C). Other transcription factors, e.g., Smad2/3, did not show the change at the protein level, and served as negative controls (Fig. 8B, C). Consistent with the above results that astrocyte activation was independent of microglia, the phosphorylated STAT3 level did not change even after microglia depletion (Fig. 8D, E), which suggested STAT3 upregulation was independent of microglia and might mainly come from activated astrocytes.

## Discussion

Our group and others have reported significant losses of brain sulfatide content in early pre-clinical stages of AD cases and animal models, which occur in an ApoE-isoform dependent manner and strongly correlate with the onset and severity of Aβ deposition [30–40]. Notably, losses of brain sulfatide levels in the early pre-clinical stages of AD are not accompanied by decreases of other major myelin lipid classes [30–34], except for plasmalogens [68]. This early and specific alteration of myelin in AD is consistent with multiple lines of evidence suggesting that myelin impairment may play an important role in AD pathology [69]. Despite this, AD is primarily considered a grey matter disorder and is certainly not a classical demyelinating disease [70, 71]. On the other hand, large-scale human studies have linked lipid metabolism-related genes with AD pathogenesis [6, 7], which is consistent with the intriguing fact that the brain is the richest organ in terms of lipid content and diversity [72]. However, there are relatively fewer studies focusing on the mechanisms regarding how specific lipids affect AD pathogenesis.

The major goal of this study was to understand the impact and consequences of adult-onset AD-like sulfatide deficiency on brain homeostasis and cognitive function. To accomplish this goal, we established an inducible myelinating cell-specific CST KO mouse model and halted sulfatide biosynthesis after myelin development/maturation, mimicking the type of sulfatide losses that occur in AD brains, and administered tamoxifen at 3–4 mo of age, when their myelin is largely formed and they already contain abundant matured oligodendrocytes [58]. As anticipated from previous findings reporting slow sulfatide turnover rates in the brain [7, 73], CST cKO mice displayed sluggish but progressive sulfatide losses in the CNS. Consistent with *Plp1* expression patterns, PNS sulfatide content was not significantly affected in the conditions examined. Unlike classic demyelination, moderate post-developmental sulfatide losses had no major impact on overall oligodendrocyte/myelin homeostasis or cell death after 9 mo post-induction assessed at the lipid, RNA, and/or protein levels.

Many AD risk genes are reported to be primarily or exclusively expressed in microglia (e.g., *Trem2*, *Cd33*, *Cr1, C1q*) [74–76] or astrocytes(e.g., *Apoe*, *Clu*, *Fermt2*, *Iqck*, *Agfg2*, *Scara3*) [6, 77–79] which are the two most important resident immune cells in CNS. Recent massive GWAS [6, 7], integrative network-based analysis, and single-cell transcriptomic studies have revealed AD immune/microglial networks [11] and disease-associated microglia/astrocytes in late-onset AD [12, 13, 16]. Surprisingly, our gene expression profiling, immunoblotting, and immunofluorescence studies revealed that adult-onset mild CNS myelin sulfatide deficiency was sufficient to result in chronic AD-like neuroinflammation with the activation of DAAs and DAMs in the absence of amyloid-β and tau pathologies. Multiple microglia and astrocyte-related AD risk genes (*Apoe*, *Trem2*, *Cd33*, *Mmp12,* and *Spi1*), AD strongly associated genes (*Tyrobp*, *Dock* and *Fcer1g*) were significantly upregulated. AD-related gene networks were consistently upregulated, including an early and strong activation of cytokine pathway (e.g., *Ccl3*, *Ccl4*, *Csf3r*, *Osmr*) and complement pathways (e.g., *C1qc*, *C1qa*, *C1qb*, *C3*, *C3ar1*); similarly, the Fc pathway was also heavily activated (e.g., *Fcgr1*, *Fcgr2b*, *Dock*), although at subsequent stages. All these results are quite similar with the expression profile results from isolated human AD cells [11].

*Apoe4* is the strongest AD risk gene. Although a lot of studies have been done to reveal its functions in amyloid-β peptide aggregation and clearance, tau neurofibrillary degeneration, microglia and astrocyte responses, and blood-brain barrier disruption [21, 22], meanwhile, ApoE is the major extracellular lipid carrier in the central nervous system (CNS) [23] and ApoE4 has the strongest binding capability to sulfatide than other ApoE allele [23]. In this study, we demonstrated that sulfatide deficiency on its own (in the absence of Aβ or tau pathologies) is sufficient to upregulate ApoE expression, which seems to occur as a consequence of astroglial, but not microglial activation, linking astrocyte ApoE with myelin sulfatide metabolism. This result is important because up-regulation of *Apoe* gene expression has been consistently reported in both AD animal models and human tissue [13, 22], but its modulation by the loss of a specific myelin lipid has never been described even though ApoE is one of the major lipid transporters in the CNS. Combined with our previous results demonstrating that CNS sulfatide metabolism is mediated by ApoE [23], our results suggest a positive feedback mechanism between sulfatide losses and subsequent ApoE overexpression by inducing astrocyte activation.

Although our previous results demonstrated that ApoE is required to bring down brain sulfatide levels [3], and ApoE overexpression was significantly increased in the CNS of CST KO as early as 2 months of age (Fig. 4B), surprisingly, brain genetic profiles of CST/ApoE double KO mice and CST KO mice of 3-mo old were highly overlapping, particularly regarding neuroinflammation signatures. This observation suggests that sulfatide deficiency can induce microgliosis and astrogliosis in an ApoE-independent manner, and that ApoE appears to be upstream of sulfatide loss but not directly involved in sulfatide deficiency-induced glia activation. It is important to mention that CST KO and CST/ApoE double KO can not mimic the age-dependent sulfatide loss, but can still be used to study the regulation among sulfatide deficiency, ApoE, and their associated neuroinflammation. Considering that sulfatide transport within the CNS is severely reduced (if not completely abolished) in ApoE deficient mice, leading to a significant accumulation of sulfatide [23], the observation that ApoE KO mice do not display gliosis implies that the loss of sulfatide within myelinating oligodendrocytes, instead of its impaired transport to other CNS cell types, is what drives neuroinflammation in sulfatide deficient mice. Putting together our latest findings with those published earlier by our group and others, we propose a model where accelerated ApoE-mediated sulfatide transport in AD, a process mediated by multiple factors including Aβ, ApoE4, increased cellular debris, and/or aging, leads to early sulfatide deficiency, which in turn triggers a neuroinflammatory response that is driven by the loss of myelin sulfatide but not through classic demyelination. Notably, recent mass spectrometry imaging studies have revealed dramatic sulfatide losses within amyloid plaques [80], where ApoE is known to aggregate, a region surrounded by reactive astrocytes and microglia, further supporting our model. Our gene expression profiling studies provided important insights into the mechanism underlying sulfatide-deficiency-induced neuroinflammation.

As far as how microglia and astrocyte were activated in myelin sulfatide deficient CNS, we didn’t find significantly altered expression of genes involved in Toll-like receptor or nucleotide-binding domain leucine-rich repeat-containing receptor pathways (e.g., *Tlr2*, *Tlr4*, *Tl*r7, *Nlrp3*, *Nod1*, *Myd88*) in the CNS of sulfatide deficient mice, which are typical pathways activated by endogenous danger signals, including cell death. It suggested that neuroinflammation after sulfatide deficiency was not induced by oligodendrocyte or cell death. Consistently, we did not find signs of cell death in the CNS of sulfatide deficient mice. On the other side, the crosstalk/interaction between oligodendrocytes, astrocytes, and microglia is well-established [81], and sulfatides are enriched within the outer membrane leaflet. Our ultrastructural images displayed numerous astrocytic processes around and in direct contact with sulfatide-deficient myelin sheaths, and our immunofluorescence studies revealed that astrogliosis and microgliosis were particularly prominent within myelin-containing regions. Furthermore, TREM2 has been shown to interact with lipidic components of myelin, including sulfatide, referred to as a lipid sensor essential in promoting microglial activation in response to insults to the white matter [23, 47, 82]. Sulfatide also directly interacts with extracellular matrix (ECM) proteins like laminin [83, 84] and tenascin [85, 86]. In addition, we also found PU.1/SPI1, STAT3 and C/EBPβ transcription factors were upregulated. SPI1 is one of the transcription factors of TREM2 expression, and TREM2 can also form positive feedback with SPI1 [87]. Totally, it is possible glial cells may sense the lack of sulfatide within the myelin sheath through interactions between glial processes and the myelin although unraveling the molecular players underlying this interaction requires future studies,

We also found that sulfatide deficiency was sufficient to disrupt both spatial and non-spatial memory. As spatial memory depends on the integrity of the hippocampus [88, 89] and recognition memory also involves the hippocampus [88], these results suggest that sulfatide deficiency leads to impaired hippocampal function. On the other hand, we did not find any sign of cell death, consistent with the fact that endogenous danger signals did not seem to be activated in sulfatide deficient conditions. Further studies are needed to clarify whether sulfatide deficiency causes abnormal conductivity explaining cognitive and memory dysfunctions [90, 91] or if only chronic neuroinflammation accelerates neurodegeneration in sulfatide deficient CNS.

## Conclusion

**Fig. 9:**
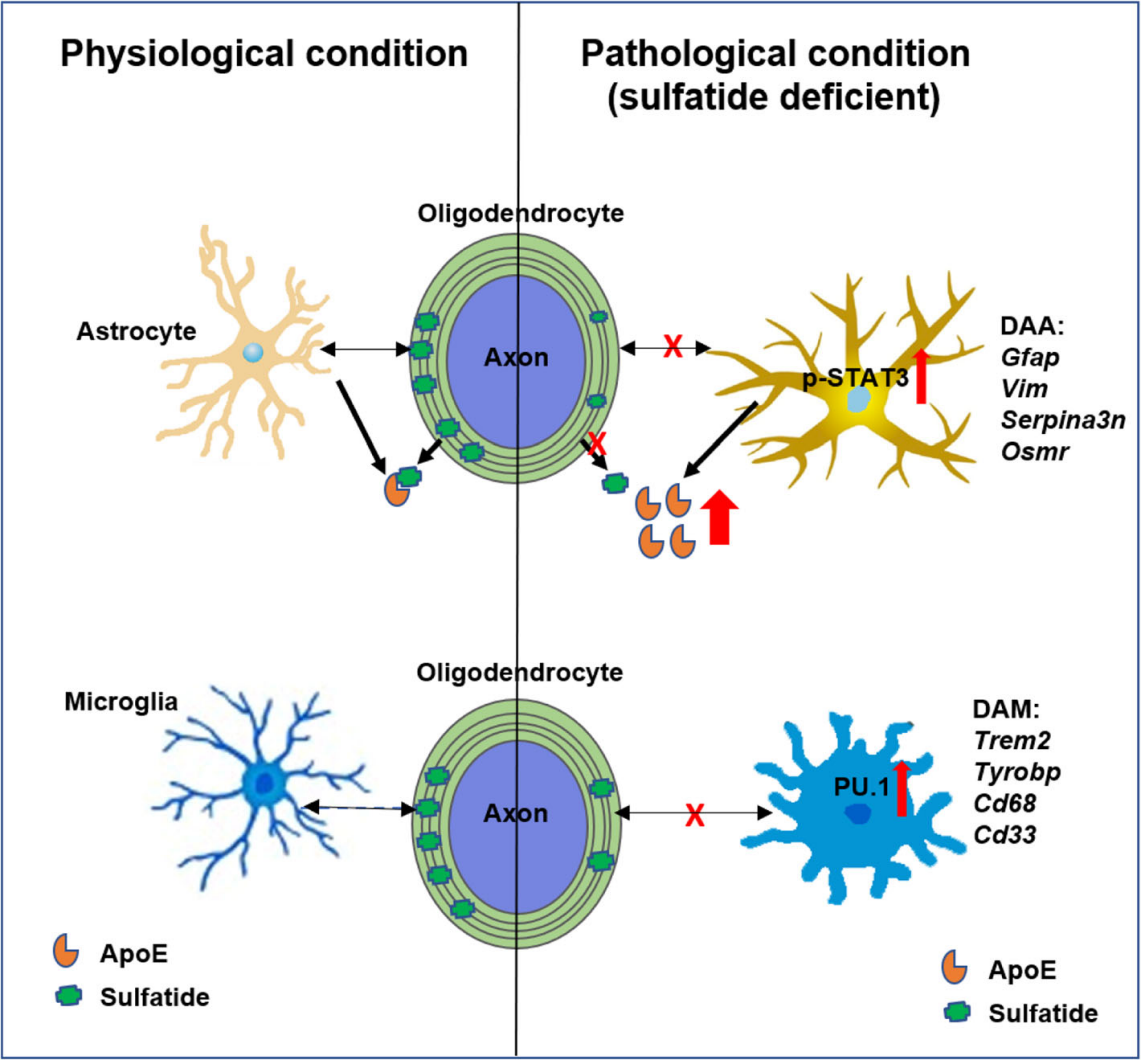
The schematic summary diagram showing the relationship between sulfatide loss, ApoE overexpression and the sulfatide deficiency caused AD-like neuroinflammation

To the best of our knowledge, this is the first study to demonstrate that brain myelin sulfatide deficiency, an early and highly specific AD metabolic abnormality driven by ApoE and accelerated by Aβ accumulation and many other factors, is sufficient to induce AD-like neuroinflammation characterized by strong activation of DAAs and DAMs, cognitive decline, and astroglial ApoE upregulation (Fig. 9).

## Supplementary Information

**Additional file 1:** Supplementary figures and figure legends.

**Additional file 2:** Original western blot figures.

## Data Availability

The datasets used and/or analyzed during the current study are available from the corresponding author on reasonable request.

## Abbreviations

Aβ: amyloid-beta
AD: Alzheimer’s disease
CNS: central nervous system
GWAS: genome-wide association studies
DAMs: disease-associated microglia
DAAs: disease-associated astrocytes
PC: phosphatidylcholine
PS: phosphatidylserine
MWM: Morris water maze
NOR: novel object recognition
GO: Gene Ontology
DEGs: differentially expressed genes
PCA: Principal component analysis
MALDI-MS: matrix-assisted laser desorption/ionization mass spectrometry
ECM: extracellular matrix

## References

[1] Cummings J, Ritter A, Zhong K (2018). Clinical trials for disease-modifying therapies in Alzheimer's disease: a primer, lessons learned, and a blueprint for the future. J Alzheimers Dis.

[2] Heneka MT, Carson MJ, Khoury JE, Landreth GE, Brosseron F, Feinstein DL, Jacobs AH, Wyss-Coray T, Vitorica J, Ransohoff RM, Herrup K, Frautschy SA, Finsen B, Brown GC, Verkhratsky A, Yamanaka K, Koistinaho J, Latz E, Halle A, Petzold GC, Town T, Morgan D, Shinohara ML, Perry VH, Holmes C, Bazan NG, Brooks DJ, Hunot S, Joseph B, Deigendesch N, Garaschuk O, Boddeke E, Dinarello CA, Breitner JC, Cole GM, Golenbock DT, Kummer MP (2015). Neuroinflammation in Alzheimer's disease. Lancet Neurol.

[3] Kaur D, Sharma V, Deshmukh R (2019). Activation of microglia and astrocytes: a roadway to neuroinflammation and Alzheimer's disease. Inflammopharmacology.

[4] Newcombe EA, Camats-Perna J, Silva ML, Valmas N, Huat TJ, Medeiros R (2018). Inflammation: the link between comorbidities, genetics, and Alzheimer's disease. J Neuroinflammation.

[5] Rajendran L, Paolicelli RC (2018). Microglia-mediated synapse loss in Alzheimer's disease. J Neurosci.

[6] Kunkle BW (2019). Genetic meta-analysis of diagnosed Alzheimer's disease identifies new risk loci and implicates Abeta, tau, immunity and lipid processing. Nat Genet.

[7] Jansen IE, Savage JE, Watanabe K, Bryois J, Williams DM, Steinberg S, Sealock J, Karlsson IK, Hägg S, Athanasiu L, Voyle N, Proitsi P, Witoelar A, Stringer S, Aarsland D, Almdahl IS, Andersen F, Bergh S, Bettella F, Bjornsson S, Brækhus A, Bråthen G, de Leeuw C, Desikan RS, Djurovic S, Dumitrescu L, Fladby T, Hohman TJ, Jonsson PV, Kiddle SJ, Rongve A, Saltvedt I, Sando SB, Selbæk G, Shoai M, Skene NG, Snaedal J, Stordal E, Ulstein ID, Wang Y, White LR, Hardy J, Hjerling-Leffler J, Sullivan PF, van der Flier WM, Dobson R, Davis LK, Stefansson H, Stefansson K, Pedersen NL, Ripke S, Andreassen OA, Posthuma D (2019). Genome-wide meta-analysis identifies new loci and functional pathways influencing Alzheimer's disease risk. Nat Genet.

[8] Raghavan NS, Brickman AM, Andrews H, Manly JJ, Schupf N, Lantigua R, Wolock CJ, Kamalakaran S, Petrovski S, Tosto G, Vardarajan BN, Goldstein DB, Mayeux R, The Alzheimer's Disease Sequencing Project (2018). Whole-exome sequencing in 20,197 persons for rare variants in Alzheimer's disease. Ann Clin Transl Neurol.

[9] Bis JC, et al. Whole exome sequencing study identifies novel rare and common Alzheimer's-associated variants involved in immune response and transcriptional regulation. Mol Psychiatry. 2020;25:1859–75. 10.1038/s41380-018-0112-7. 10.1038/s41380-018-0112-7 PMC6375806 30108311

[10] Campion D, Charbonnier C, Nicolas G (2019). SORL1 genetic variants and Alzheimer disease risk: a literature review and meta-analysis of sequencing data. Acta Neuropathol.

[11] Zhang B, Gaiteri C, Bodea LG, Wang Z, McElwee J, Podtelezhnikov AA, Zhang C, Xie T, Tran L, Dobrin R, Fluder E, Clurman B, Melquist S, Narayanan M, Suver C, Shah H, Mahajan M, Gillis T, Mysore J, MacDonald ME, Lamb JR, Bennett DA, Molony C, Stone DJ, Gudnason V, Myers AJ, Schadt EE, Neumann H, Zhu J, Emilsson V (2013). Integrated systems approach identifies genetic nodes and networks in late-onset Alzheimer's disease. Cell.

[12] Grubman A, Chew G, Ouyang JF, Sun G, Choo XY, McLean C, Simmons RK, Buckberry S, Vargas-Landin DB, Poppe D, Pflueger J, Lister R, Rackham OJL, Petretto E, Polo JM (2019). A single-cell atlas of entorhinal cortex from individuals with Alzheimer's disease reveals cell-type-specific gene expression regulation. Nat Neurosci.

[13] Mathys H, Davila-Velderrain J, Peng Z, Gao F, Mohammadi S, Young JZ, Menon M, He L, Abdurrob F, Jiang X, Martorell AJ, Ransohoff RM, Hafler BP, Bennett DA, Kellis M, Tsai LH (2019). Single-cell transcriptomic analysis of Alzheimer's disease. Nature.

[14] Bryois J (2020). Genetic identification of cell types underlying brain complex traits yields insights into the etiology of Parkinson's disease. Nat Genet.

[15] Keren-Shaul H, Spinrad A, Weiner A, Matcovitch-Natan O, Dvir-Szternfeld R, Ulland TK, David E, Baruch K, Lara-Astaiso D, Toth B, Itzkovitz S, Colonna M, Schwartz M, Amit I (2017). A unique microglia type associated with restricting development of Alzheimer's disease. Cell.

[16] Habib N, McCabe C, Medina S, Varshavsky M, Kitsberg D, Dvir-Szternfeld R, Green G, Dionne D, Nguyen L, Marshall JL, Chen F, Zhang F, Kaplan T, Regev A, Schwartz M (2020). Disease-associated astrocytes in Alzheimer's disease and aging. Nat Neurosci.

[17] Masuda T, Tsuda M, Yoshinaga R, Tozaki-Saitoh H, Ozato K, Tamura T, Inoue K (2012). IRF8 is a critical transcription factor for transforming microglia into a reactive phenotype. Cell Rep.

[18] Ben Haim L, Ceyzeriat K, Carrillo-de Sauvage MA, Aubry F, Auregan G, Guillermier M, Ruiz M, Petit F, Houitte D, Faivre E, Vandesquille M, Aron-Badin R, Dhenain M, Deglon N, Hantraye P, Brouillet E, Bonvento G, Escartin C (2015). The JAK/STAT3 pathway is a common inducer of astrocyte reactivity in Alzheimer's and Huntington's diseases. J Neurosci.

[19] Smith AM, Gibbons HM, Oldfield RL, Bergin PM, Mee EW, Faull RLM, Dragunow M (2013). The transcription factor PU.1 is critical for viability and function of human brain microglia. Glia.

[20] Tansey KE, Cameron D, Hill MJ (2018). Genetic risk for Alzheimer's disease is concentrated in specific macrophage and microglial transcriptional networks. Genome Med.

[21] Serrano-Pozo A, Das S, Hyman BT (2021). APOE and Alzheimer's disease: advances in genetics, pathophysiology, and therapeutic approaches. Lancet Neurol.

[22] Gottschalk, W.K., et al., The Role of Upregulated APOE in Alzheimer's Disease Etiology*.* J Alzheimers Dis Parkinsonism, 2016. **6**(1), DOI: 10.4172/2161-0460.1000209. 10.4172/2161-0460.1000209 PMC4836841 27104063

[23] Han X, Cheng H, Fryer JD, Fagan AM, Holtzman DM (2003). Novel role for apolipoprotein E in the central nervous system. Modulation of sulfatide content. J Biol Chem.

[24] Xu Q, Bernardo A, Walker D, Kanegawa T, Mahley RW, Huang Y (2006). Profile and regulation of apolipoprotein E (ApoE) expression in the CNS in mice with targeting of green fluorescent protein gene to the ApoE locus. J Neurosci.

[25] Han X (2007). Neurolipidomics: challenges and developments. Front Biosci.

[26] Mitew S, Kirkcaldie MTK, Halliday GM, Shepherd CE, Vickers JC, Dickson TC (2010). Focal demyelination in Alzheimer's disease and transgenic mouse models. Acta Neuropathol.

[27] Carmeli C, Donati A, Antille V, Viceic D, Ghika J, von Gunten A, Clarke S, Meuli R, Frackowiak RS, Knyazeva MG (2013). Demyelination in mild cognitive impairment suggests progression path to Alzheimer's disease. PLoS One.

[28] Lau SF, Cao H, Fu AKY, Ip NY (2020). Single-nucleus transcriptome analysis reveals dysregulation of angiogenic endothelial cells and neuroprotective glia in Alzheimer's disease. Proc Natl Acad Sci U S A.

[29] Bartzokis G (2011). Alzheimer's disease as homeostatic responses to age-related myelin breakdown. Neurobiol Aging.

[30] Han X, Fagan AM, Cheng H, Morris JC, Xiong C, Holtzman DM (2003). Cerebrospinal fluid sulfatide is decreased in subjects with incipient dementia. Ann Neurol.

[31] Han X, M. Holtzman D, W. McKeel D, Kelley J, Morris JC (2002). Substantial sulfatide deficiency and ceramide elevation in very early Alzheimer's disease: potential role in disease pathogenesis. J Neurochem.

[32] Cheng H, Wang M, Li JL, Cairns NJ, Han X (2013). Specific changes of sulfatide levels in individuals with pre-clinical Alzheimer's disease: an early event in disease pathogenesis. J Neurochem.

[33] Han X (2005). Lipid alterations in the earliest clinically recognizable stage of Alzheimer's disease: implication of the role of lipids in the pathogenesis of Alzheimer's disease. Curr Alzheimer Res.

[34] Irizarry MC (2003). A turn of the sulfatide in Alzheimer's disease. Ann Neurol.

[35] Cheng H, Zhou Y, Holtzman DM, Han X (2010). Apolipoprotein E mediates sulfatide depletion in animal models of Alzheimer's disease. Neurobiol Aging.

[36] Hong JH, Kang JW, Kim DK, Baik SH, Kim KH, Shanta SR, Jung JH, Mook-Jung I, Kim KP (2016). Global changes of phospholipids identified by MALDI imaging mass spectrometry in a mouse model of Alzheimer's disease. J Lipid Res.

[37] Han X (2010). The pathogenic implication of abnormal interaction between apolipoprotein E isoforms, amyloid-beta peptides, and sulfatides in Alzheimer's disease. Mol Neurobiol.

[38] Kaya I, Brinet D, Michno W, Syvänen S, Sehlin D, Zetterberg H, Blennow K, Hanrieder J (2017). Delineating amyloid plaque associated neuronal sphingolipids in transgenic Alzheimer's disease mice (tgArcSwe) using MALDI imaging mass spectrometry. ACS Chem Neurosci.

[39] Kaya I (2020). Brain region-specific amyloid plaque-associated myelin lipid loss, APOE deposition and disruption of the myelin sheath in familial Alzheimer's disease mice. J Neurochem.

[40] Wallin A, Gottfries CG, Karlsson I, Svennerholm L (1989). Decreased myelin lipids in Alzheimer's disease and vascular dementia. Acta Neurol Scand.

[41] Carlo AS, Gustafsen C, Mastrobuoni G, Nielsen MS, Burgert T, Hartl D, Rohe M, Nykjaer A, Herz J, Heeren J, Kempa S, Petersen CM, Willnow TE (2013). The pro-neurotrophin receptor sortilin is a major neuronal apolipoprotein E receptor for catabolism of amyloid-beta peptide in the brain. J Neurosci.

[42] Carlo AS (2013). Sortilin, a novel APOE receptor implicated in Alzheimer disease. Prion.

[43] Andersson CH, Hansson O, Minthon L, Andreasen N, Blennow K, Zetterberg H, Skoog I, Wallin A, Nilsson S, Kettunen P (2016). A genetic variant of the Sortilin 1 gene is associated with reduced risk of Alzheimer's disease. J Alzheimers Dis.

[44] Stoeck K, Psychogios MN, Ohlenbusch A, Steinfeld R, Schmidt J (2016). Late-onset metachromatic Leukodystrophy with early onset dementia associated with a novel missense mutation in the arylsulfatase a gene. J Alzheimers Dis.

[45] Blue EE, Bis JC, Dorschner MO, Tsuang DW, Barral SM, Beecham G, Below JE, Bush WS, Butkiewicz M, Cruchaga C, DeStefano A, Farrer LA, Goate A, Haines J, Jaworski J, Jun G, Kunkle B, Kuzma A, Lee JJ, Lunetta KL, Ma Y, Martin E, Naj A, Nato AQ, Navas P, Nguyen H, Reitz C, Reyes D, Salerno W, Schellenberg GD, Seshadri S, Sohi H, Thornton TA, Valadares O, van Duijn C, Vardarajan BN, Wang LS, Boerwinkle E, Dupuis J, Pericak-Vance MA, Mayeux R, Wijsman EM, on behalf of the Alzheimer’s Disease Sequencing Project (2018). Genetic variation in genes underlying diverse dementias may explain a small proportion of cases in the Alzheimer's disease sequencing project. Dement Geriatr Cogn Disord.

[46] Guerreiro R, Wojtas A, Bras J, Carrasquillo M, Rogaeva E, Majounie E, Cruchaga C, Sassi C, Kauwe JS, Younkin S, Hazrati L, Collinge J, Pocock J, Lashley T, Williams J, Lambert JC, Amouyel P, Goate A, Rademakers R, Morgan K, Powell J, St George-Hyslop P, Singleton A, Hardy J, Alzheimer Genetic Analysis Group (2013). TREM2 variants in Alzheimer's disease. N Engl J Med.

[47] Wang Y, Cella M, Mallinson K, Ulrich JD, Young KL, Robinette ML, Gilfillan S, Krishnan GM, Sudhakar S, Zinselmeyer BH, Holtzman DM, Cirrito JR, Colonna M (2015). TREM2 lipid sensing sustains the microglial response in an Alzheimer's disease model. Cell.

[48] Whitehead JC, Hildebrand BA, Sun M, Rockwood MR, Rose RA, Rockwood K, Howlett SE (2014). A clinical frailty index in aging mice: comparisons with frailty index data in humans. J Gerontol A Biol Sci Med Sci.

[49] Morris R (1984). Developments of a water-maze procedure for studying spatial learning in the rat. J Neurosci Methods.

[50] Palavicini JP, Wang C, Chen L, Ahmar S, Higuera JD, Dupree JL, Han X (2016). Novel molecular insights into the critical role of sulfatide in myelin maintenance/function. J Neurochem.

[51] Cheng H, Jiang X, Han X (2007). Alterations in lipid homeostasis of mouse dorsal root ganglia induced by apolipoprotein E deficiency: a shotgun lipidomics study. J Neurochem.

[52] Yang K, Cheng H, Gross RW, Han X (2009). Automated lipid identification and quantification by multidimensional mass spectrometry-based shotgun lipidomics. Anal Chem.

[53] Wang M, Wang C, Han X (2016). Selection of internal standards for accurate quantification of complex lipid species in biological extracts by electrospray ionization mass spectrometry-what, how and why?. Mass Spectrom Rev.

[54] Wang J, Qiu S, Chen S, Xiong C, Liu H, Wang J, Zhang N, Hou J, He Q, Nie Z (2015). MALDI-TOF MS imaging of metabolites with a N-(1-naphthyl) ethylenediamine dihydrochloride matrix and its application to colorectal cancer liver metastasis. Anal Chem.

[55] Marcus J, Honigbaum S, Shroff S, Honke K, Rosenbluth J, Dupree JL (2006). Sulfatide is essential for the maintenance of CNS myelin and axon structure. Glia.

[56] Coetzee T, Fujita N, Dupree J, Shi R, Blight A, Suzuki K, Suzuki K, Popko B (1996). Myelination in the absence of galactocerebroside and sulfatide: normal structure with abnormal function and regional instability. Cell.

[57] Doerflinger NH, Macklin WB, Popko B (2003). Inducible site-specific recombination in myelinating cells. Genesis.

[58] Hughes EG, Orthmann-Murphy JL, Langseth AJ, Bergles DE (2018). Myelin remodeling through experience-dependent oligodendrogenesis in the adult somatosensory cortex. Nat Neurosci.

[59] Preuss C (2020). A novel systems biology approach to evaluate mouse models of late-onset Alzheimer's disease. Mol Neurodegener.

[60] Huang Y, Zhou W, Zhang Y (2012). Bright lighting conditions during testing increase thigmotaxis and impair water maze performance in BALB/c mice. Behav Brain Res.

[61] Fitzner D, Bader JM, Penkert H, Bergner CG, Su M, Weil MT, Surma MA, Mann M, Klose C, Simons M (2020). Cell-type- and brain-region-resolved mouse brain Lipidome. Cell Rep.

[62] Chen EY, Tan CM, Kou Y, Duan Q, Wang Z, Meirelles GV, Clark NR, Ma’ayan A (2013). Enrichr: interactive and collaborative HTML5 gene list enrichment analysis tool. BMC Bioinformatics.

[63] Krasemann S, Madore C, Cialic R, Baufeld C, Calcagno N, el Fatimy R, Beckers L, O’Loughlin E, Xu Y, Fanek Z, Greco DJ, Smith ST, Tweet G, Humulock Z, Zrzavy T, Conde-Sanroman P, Gacias M, Weng Z, Chen H, Tjon E, Mazaheri F, Hartmann K, Madi A, Ulrich JD, Glatzel M, Worthmann A, Heeren J, Budnik B, Lemere C, Ikezu T, Heppner FL, Litvak V, Holtzman DM, Lassmann H, Weiner HL, Ochando J, Haass C, Butovsky O (2017). The TREM2-APOE pathway drives the transcriptional phenotype of dysfunctional microglia in neurodegenerative diseases. Immunity.

[64] Kang SS, Ebbert MTW, Baker KE, Cook C, Wang X, Sens JP, Kocher JP, Petrucelli L, Fryer JD (2018). Microglial translational profiling reveals a convergent APOE pathway from aging, amyloid, and tau. J Exp Med.

[65] Elmore MR (2014). Colony-stimulating factor 1 receptor signaling is necessary for microglia viability, unmasking a microglia progenitor cell in the adult brain. Neuron.

[66] Rice RA, Spangenberg EE, Yamate-Morgan H, Lee RJ, Arora RPS, Hernandez MX, Tenner AJ, West BL, Green KN (2015). Elimination of microglia improves functional outcomes following extensive neuronal loss in the Hippocampus. J Neurosci.

[67] Xia Y, Wang ZH, Zhang J, Liu X, Yu SP, Ye KX, et al. C/EBPbeta is a key transcription factor for APOE and preferentially mediates ApoE4 expression in Alzheimer's disease. Mol Psychiatry. 2020. 10.1038/s41380-020-00956-4. 10.1038/s41380-020-00956-4 PMC8758498 33339957

[68] Han X, Holtzman DM, McKeel DW (2001). Plasmalogen deficiency in early Alzheimer's disease subjects and in animal models: molecular characterization using electrospray ionization mass spectrometry. J Neurochem.

[69] Papuc E, Rejdak K (2020). The role of myelin damage in Alzheimer's disease pathology. Arch Med Sci.

[70] Zhang SC, Goetz BD, Carré JL, Duncan ID (2001). Reactive microglia in dysmyelination and demyelination. Glia.

[71] Love S (2006). Demyelinating diseases. J Clin Pathol.

[72] Caso F, Agosta F, Mattavelli D, Migliaccio R, Canu E, Magnani G, Marcone A, Copetti M, Falautano M, Comi G, Falini A, Filippi M (2015). White matter degeneration in atypical Alzheimer disease. Radiology.

[73] Moser HW, Moser AB, McKhann GM (1967). The dynamics of a lipidosis. Turnover of sulfatide, steroid sulfate, and polysaccharide sulfate in metachromatic leukodystrophy. Arch Neurol.

[74] Sims R (2017). Rare coding variants in PLCG2, ABI3, and TREM2 implicate microglial-mediated innate immunity in Alzheimer's disease. Nat Genet.

[75] Karch CM, Goate AM (2015). Alzheimer's disease risk genes and mechanisms of disease pathogenesis. Biol Psychiatry.

[76] Efthymiou AG, Goate AM (2017). Late onset Alzheimer's disease genetics implicates microglial pathways in disease risk. Mol Neurodegener.

[77] Lambert JC (2009). Genome-wide association study identifies variants at CLU and CR1 associated with Alzheimer's disease. Nat Genet.

[78] Seshadri S, Fitzpatrick AL, Ikram MA, DeStefano A, Gudnason V, Boada M, Bis JC, Smith AV, Carassquillo MM, Lambert JC, Harold D, Schrijvers EM, Ramirez-Lorca R, Debette S, Longstreth WT Jr, Janssens AC, Pankratz VS, Dartigues JF, Hollingworth P, Aspelund T, Hernandez I, Beiser A, Kuller LH, Koudstaal PJ, Dickson DW, Tzourio C, Abraham R, Antunez C, du Y, Rotter JI, Aulchenko YS, Harris TB, Petersen RC, Berr C, Owen MJ, Lopez-Arrieta J, Varadarajan BN, Becker JT, Rivadeneira F, Nalls MA, Graff-Radford NR, Campion D, Auerbach S, Rice K, Hofman A, Jonsson PV, Schmidt H, Lathrop M, Mosley TH, Au R, Psaty BM, Uitterlinden AG, Farrer LA, Lumley T, Ruiz A, Williams J, Amouyel P, Younkin SG, Wolf PA, Launer LJ, Lopez OL, van Duijn C, Breteler MM, CHARGE Consortium., GERAD1 Consortium., EADI1 Consortium (2010). Genome-wide analysis of genetic loci associated with Alzheimer disease. JAMA.

[79] Chapuis J (2017). Genome-wide, high-content siRNA screening identifies the Alzheimer's genetic risk factor FERMT2 as a major modulator of APP metabolism. Acta Neuropathol.

[80] Kaya I, Jennische E, Lange S, Tarik Baykal A, Malmberg P, Fletcher JS (2020). Brain region-specific amyloid plaque-associated myelin lipid loss, APOE deposition and disruption of the myelin sheath in familial Alzheimer's disease mice. J Neurochem.

[81] Domingues HS (2016). Oligodendrocyte, astrocyte, and microglia crosstalk in myelin development, damage, and repair. Front Cell Dev Biol.

[82] Poliani PL, Wang Y, Fontana E, Robinette ML, Yamanishi Y, Gilfillan S, Colonna M (2015). TREM2 sustains microglial expansion during aging and response to demyelination. J Clin Invest.

[83] Baron W, Bijlard M, Nomden A, de Jonge JC, Teunissen CE, Hoekstra D (2014). Sulfatide-mediated control of extracellular matrix-dependent oligodendrocyte maturation. Glia.

[84] Li S, Liquari P, McKee KK, Harrison D, Patel R, Lee S, Yurchenco PD (2005). Laminin-sulfatide binding initiates basement membrane assembly and enables receptor signaling in Schwann cells and fibroblasts. J Cell Biol.

[85] Pesheva P, Gloor S, Schachner M, Probstmeier R (1997). Tenascin-R is an intrinsic autocrine factor for oligodendrocyte differentiation and promotes cell adhesion by a sulfatide-mediated mechanism. J Neurosci.

[86] Shao K, Hou Q, Go ML, Duan W, Cheung NS, Feng SS, Wong KP, Yoram A, Zhang W, Huang Z, Li QT (2007). Sulfatide-tenascin interaction mediates binding to the extracellular matrix and endocytic uptake of liposomes in glioma cells. Cell Mol Life Sci.

[87] Carbajosa G, Malki K, Lawless N, Wang H, Ryder JW, Wozniak E, Wood K, Mein CA, Dobson RJB, Collier DA, O'Neill MJ, Hodges AK, Newhouse SJ (2018). Loss of Trem2 in microglia leads to widespread disruption of cell coexpression networks in mouse brain. Neurobiol Aging.

[88] Broadbent NJ, Squire LR, Clark RE (2004). Spatial memory, recognition memory, and the hippocampus. Proc Natl Acad Sci U S A.

[89] Vorhees CV, Williams MT (2014). Assessing spatial learning and memory in rodents. ILAR J.

[90] Zhao J, Bi W, Xiao S, Lan X, Cheng X, Zhang J, Lu D, Wei W, Wang Y, Li H, Fu Y, Zhu L (2019). Neuroinflammation induced by lipopolysaccharide causes cognitive impairment in mice. Sci Rep.

[91] Ali W, Ikram M, Park HY, Jo MG, Ullah R, Ahmad S, et al. Oral Administration of Alpha Linoleic Acid Rescues Abeta-Induced Glia-Mediated Neuroinflammation and Cognitive Dysfunction in C57BL/6N Mice. Cells. 2020;9(3). 10.3390/cells9030667. 10.3390/cells9030667 PMC7140708 32182943

