# Supplementary material for: Adult-onset CNS myelin sulfatide deficiency is sufficient to cause Alzheimer’s disease-like neuroinflammation and cognitive impairment
Source: Mol Neurodegener. 2021 Sep 15;16:64. doi: 10.1186/s13024-021-00488-7 (PMC8442347; doi:10.1186/s13024-021-00488-7)
Supplement: Supplementary file 1 — Additional file 1: Supplementary figures and figure legends. [file 13024_2021_488_MOESM1_ESM.docx]

**Adult-onset CNS** **myelin sulfatide deficiency is sufficient to cause** **Alzheimer’s disease-like neuroinflammation and cognitive impairment**

Shulan Qiu^1,#^, Juan Pablo Palavicini^1,2#^, Jianing Wang^1^, Nancy S. Gonzalez^1^, Sijia He^1^, Elizabeth Dustin^6^, Cheng Zou^3^, Lin Ding^1,4^, Anindita Bhattacharjee^1^, Candice E. Van Skike^1,5^, Veronica Galvan^1,5^, Jeffrey L. Dupree^6,7^, Xianlin Han^1,2,*^

^#^These authors contributed equally

^1^Barshop Institute for Longevity and Aging Studies,

University of Texas Health Science Center at San Antonio, San Antonio, Texas, 78229, USA

^2^Division of Diabetes, Department of Medicine,

University of Texas Health Science Center at San Antonio, San Antonio, Texas, 78229, USA

^3^BRC Bioinformatics Facility, Institute of Biotechnology, Cornell University, Ithaca, NY 14853, USA

^4^College of Food Science and Engineering, Ocean University of China, Qingdao, 266003, China

^5^ Department of Cellular and Integrative Physiology,

University of Texas Health Science Center at San Antonio, San Antonio, Texas, 78229, USA

^6^Department of Anatomy and Neurobiology,

Virginia Commonwealth University, Richmond, Virginia, 23284, USA

^7^Research Division, McGuire Veterans Affairs Medical Center, Richmond, Virginia, 23249, USA

Present address of Jianing Wang: State Key Lab. of Environmental & Biological Analysis, Department of Chemistry, Hong Kong Baptist University, Hongkong, China

^*^To whom correspondence should be addressed:

Xianlin Han, Ph.D.

Barshop Institute for Longevity and Aging Studies

University of Texas Health Science Center at San Antonio

4939 Charles Katz Drive

San Antonio, TX, 78229 USA

[hanx@uthscsa.edu](mailto:hanx@uthscsa.edu)

**Supplemental Figures and figure legends**


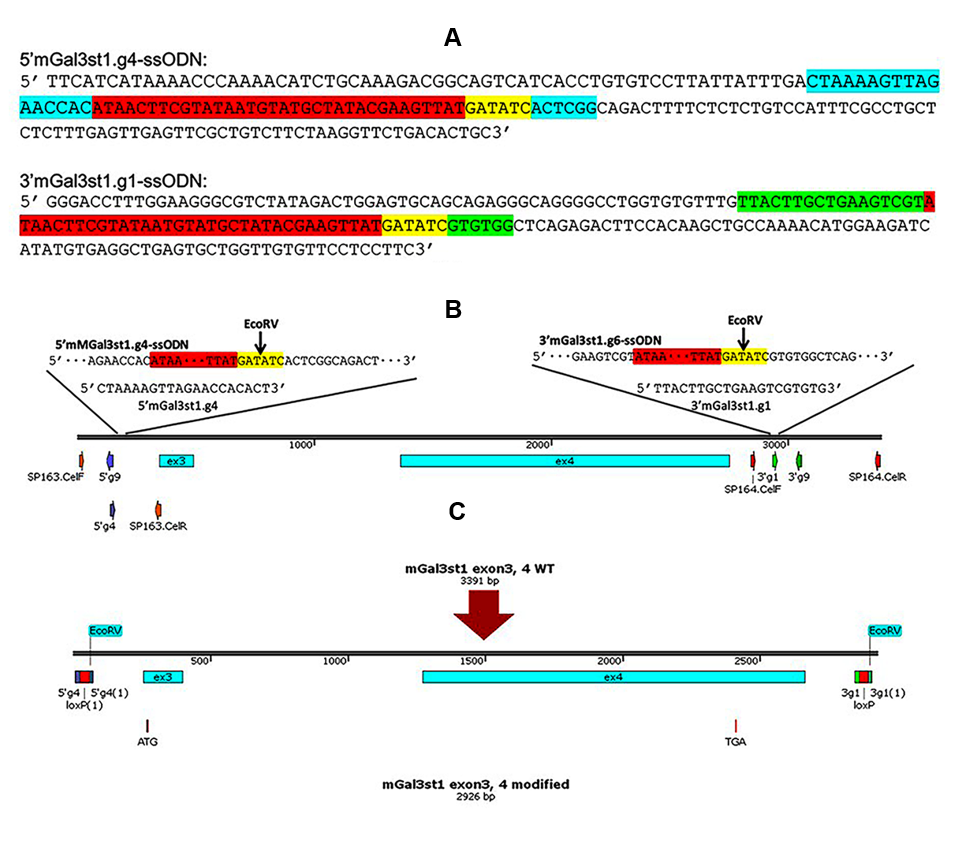


**Fig. S1. Generation of CST floxed mice.** Schematic illustration summarizing the process of generating a conditional allele at the mouse Gal3st1 (CST) gene through embryo-based homology directed repair (HDR)-mediated gene editing. (**A**) 5’-donor oligo DNA; (**B**) 3’-donor oligo DNA; (**C**) editing scheme to create CST cKO model. Legends: 5’-gRNA; EcoRV; 3’-gRNA ; loxP sequence.


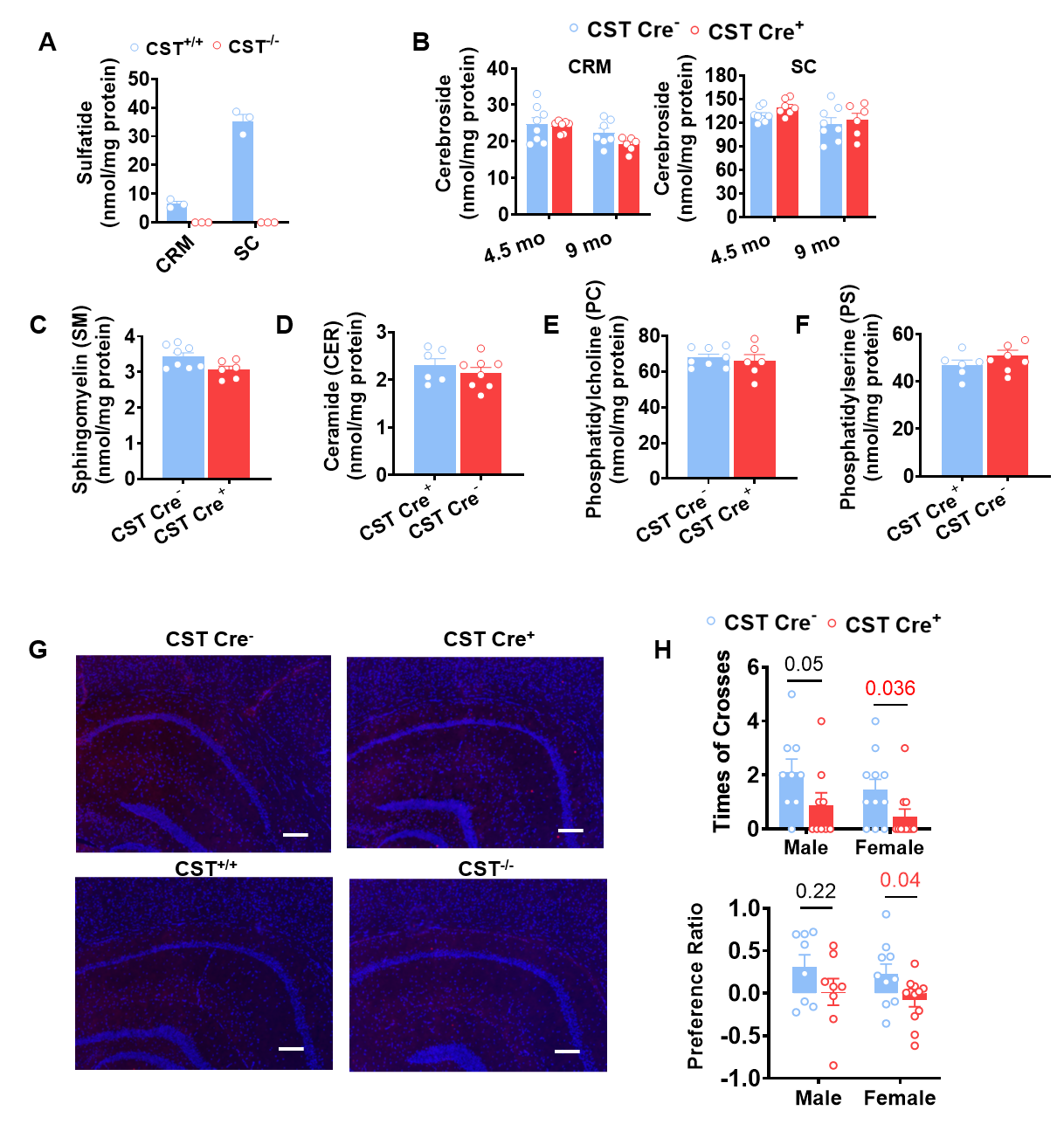


**Fig. S2. CNS-specific ST deficiency in a novel inducible myelinating glia-specific CST cKO mouse model does not have major effects on other myelin lipids, oligodendrocyte pathway, and neural cell death.** Related to Fig. 1. **(A)** The undetectable sulfatide in CST KO mouse brain compared to WT mouse confirmed the shotgun lipidomics analysis on sulfatide. **(B-G)** CRM and/or SC lipid extracts were assessed by shotgun lipidomics. **(B**)Total cerebroside. **(C)** Sphingomyelin. **(D)** Ceramide. **(E)** Phosphatidylcholine (PC). **(F)** Phosphatidylserine (PS). Similar results were obtained from SC (some data not shown). **(G)** Showing TUNEL staining on the partial hippocampus of CST cKO and KO mice. Scale bar: 200 μm. **(H)** Cross times of probe test (above) and NOR test results (below) with sex difference. **(B**) Two-way ANOVA with Bonferroni post-hoc test for multiple comparisons, n=6-8. **(C-F, H)** Two-tailed unpaired t-Test, n=6-8. Data represent the mean ± S.E.M.


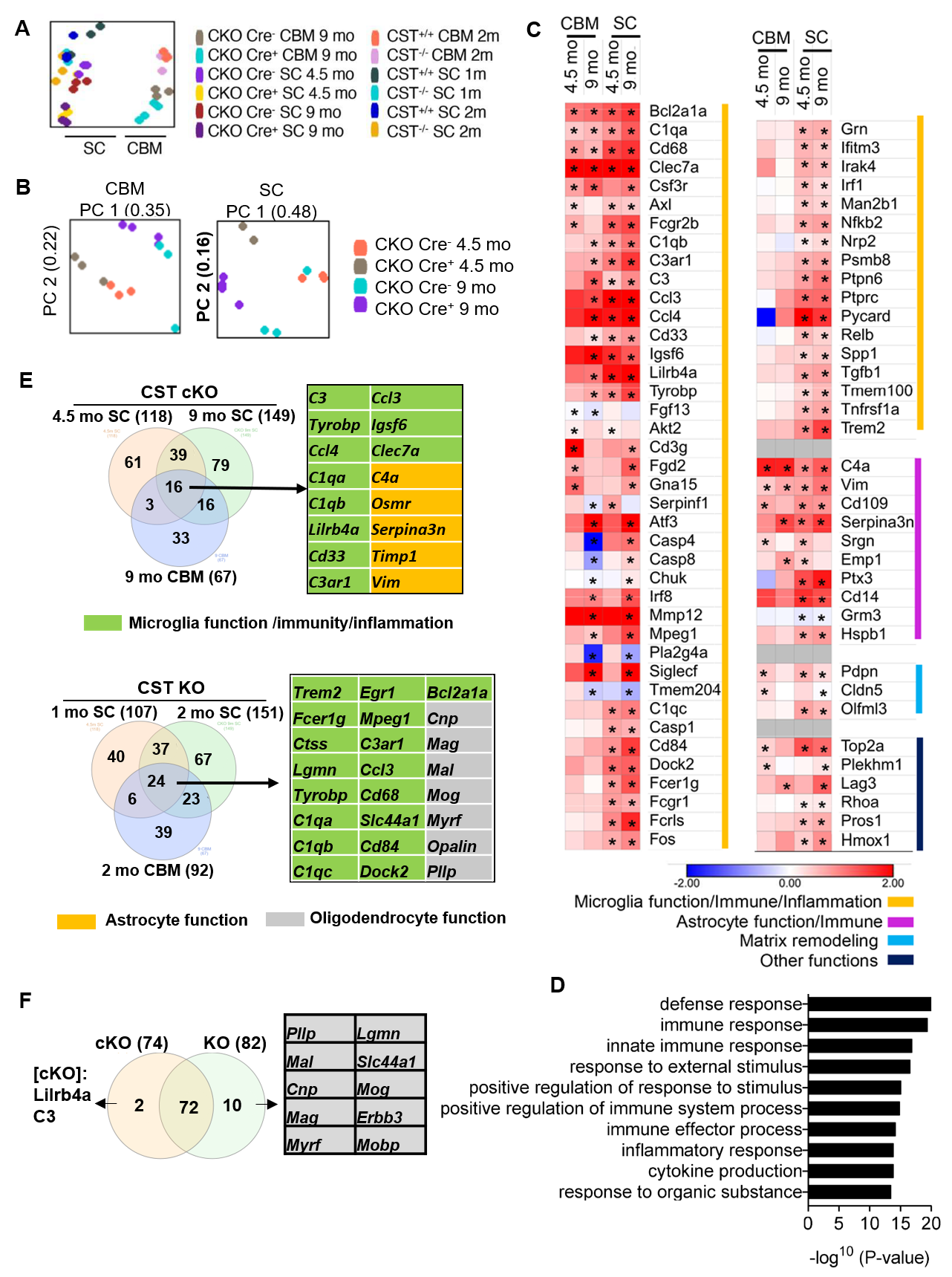


**Fig. S3. CNS sulfatide depletion induces a chronic immune/inflammatory response with microgliosis and astrogliosis.** Related to Fig. 3. **(A,B)** PCA from RNA datasets in CRM or SC of CST cKO mice with two time points (4.5 mo and 9 mo post-injection) using the NanoString mouse neuroinflammation Panel. (**C**) Heatmap displaying log2 fold changes of the 76 DEGs that were shared by at least two groups in CST cKO mice. DEGs were grouped into four major categories lines and ordered based on how many times they were significantly altered in four groups. (**D)** List of the top functions from GO analysis for the 76 shared DEGs. (**E)** Venn diagram showing the number of specific and shared DEGs in the CRM and SC between different CNS region/time point combinations from CST cKO or CST KO mice compared to their respective controls. (**F**) DEGs specific for CST cKO or CST KO mice are listed corresponding to Fig. 3C.


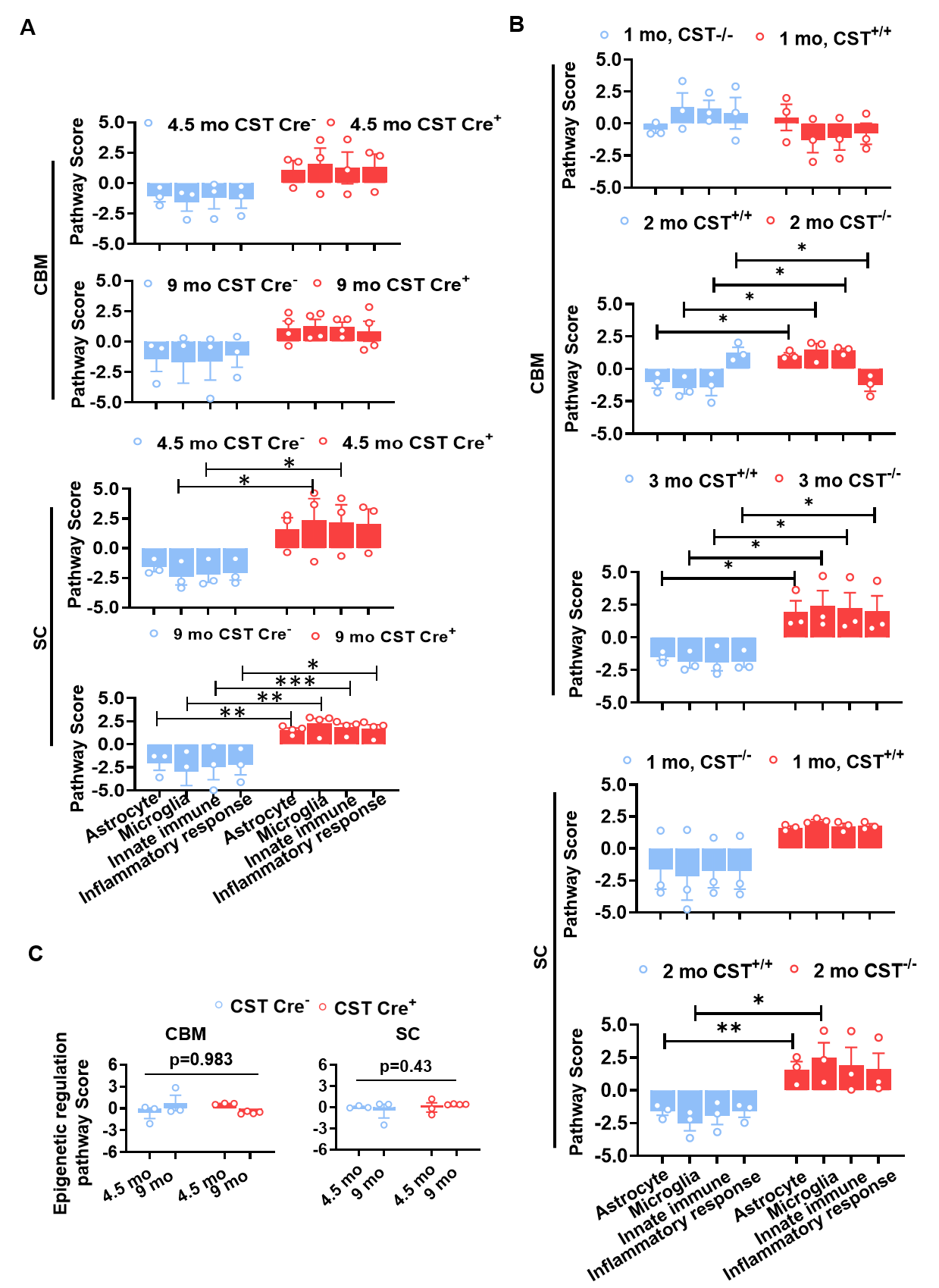


**Fig. S4. CNS sulfatide depletion induces a chronic immune/inflammatory response with microgliosis and astrogliosis.** Related to Fig. 3. **(A)** NanoString pathway score for astrocyte function, microglia function, innate immune response, and inflammatory response in the CRM and SC of CST cKO mice 4.5- and 9-mo post-injection (respective CST Cre^-^ as controls). **(B)** NanoString pathway score for astrocyte function, microglia function, innate immune response, and inflammatory signaling in the CRM and SC of CST KO mice of 1-mo, 2-mo, and 3-mo old (respective WT as controls). **(C)** Epigenetic regulation pathway score was shown as the negative control for (**A, B**). Two-way ANOVA, n=3-4. *p < 0.05, **p < 0.01. Data represent the mean ± S.E.M.


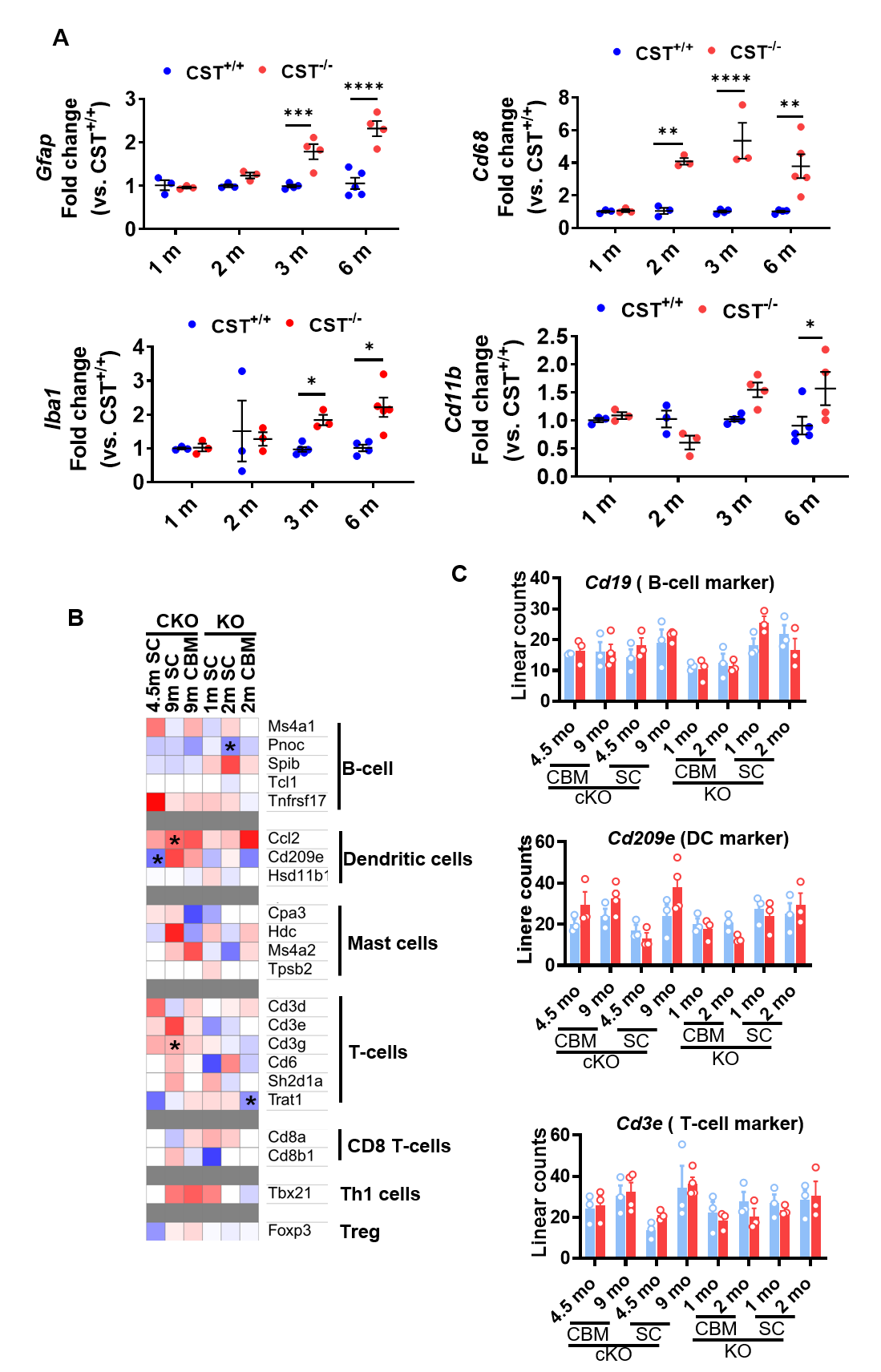


**Fig. S5. The adult-onset partial loss and embryonic-onset complete deletion of sulfatide didn’t cause the infiltration of immune cells from circulation.** Related to Fig. 3. **(A)** Real-time q-PCR result using brain stem mRNA from 1-, 2-, 3- and 6 mo-old CST^+/+^ and CST^-/-^ mice. **(B)** Heatmap displaying log2 fold changes of the cell markers of B-, Dendritic, mast, T-, CD8 T-, Th1 and Treg cells in the six groups from both CST cKO and CST KO mice. P-values are displayed within each heat map cell (*p < 0.05). (**C)** The linear counts of some specific genes to show their low and unchanged transcript copies. Data represent the mean ± S.E.M. *p < 0.05, **p < 0.01, ***p < 0.001. Data represent the mean ± S.E.M.


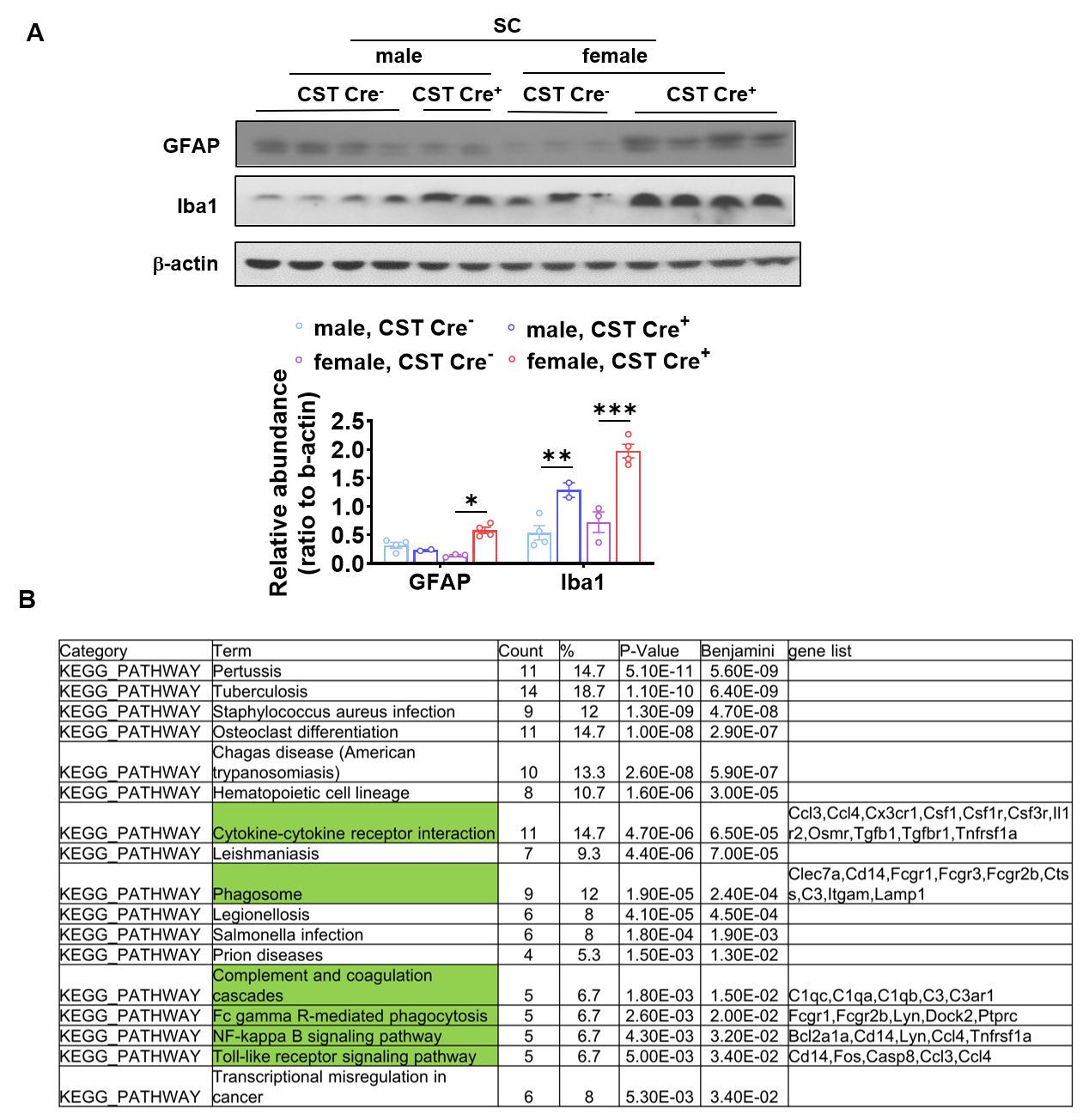


**Fig. S6. Analysis of immune/inflammation-related pathways in sulfatide deficient CNS.** Related to Fig. 4. **(A)** Western blot ananlsis of astrocyte/microglia activation-related markers with sex difference, i.e., GFAP and Iba1. Relative expression was quantified and plotted as a ratio to β-actin**. (B)** KEGG analysis for the 72 shared DEGs was performed using the Database for Annotation, Visualization and Integrated Discovery (DAVID). The top biological pathways are shown. Gene counts, p-value, and corrected p-value (Benjamini) are shown for each term. Immune/inflammation-related pathways that have been correlated with Alzheimer’s Disease were highlighted in green.


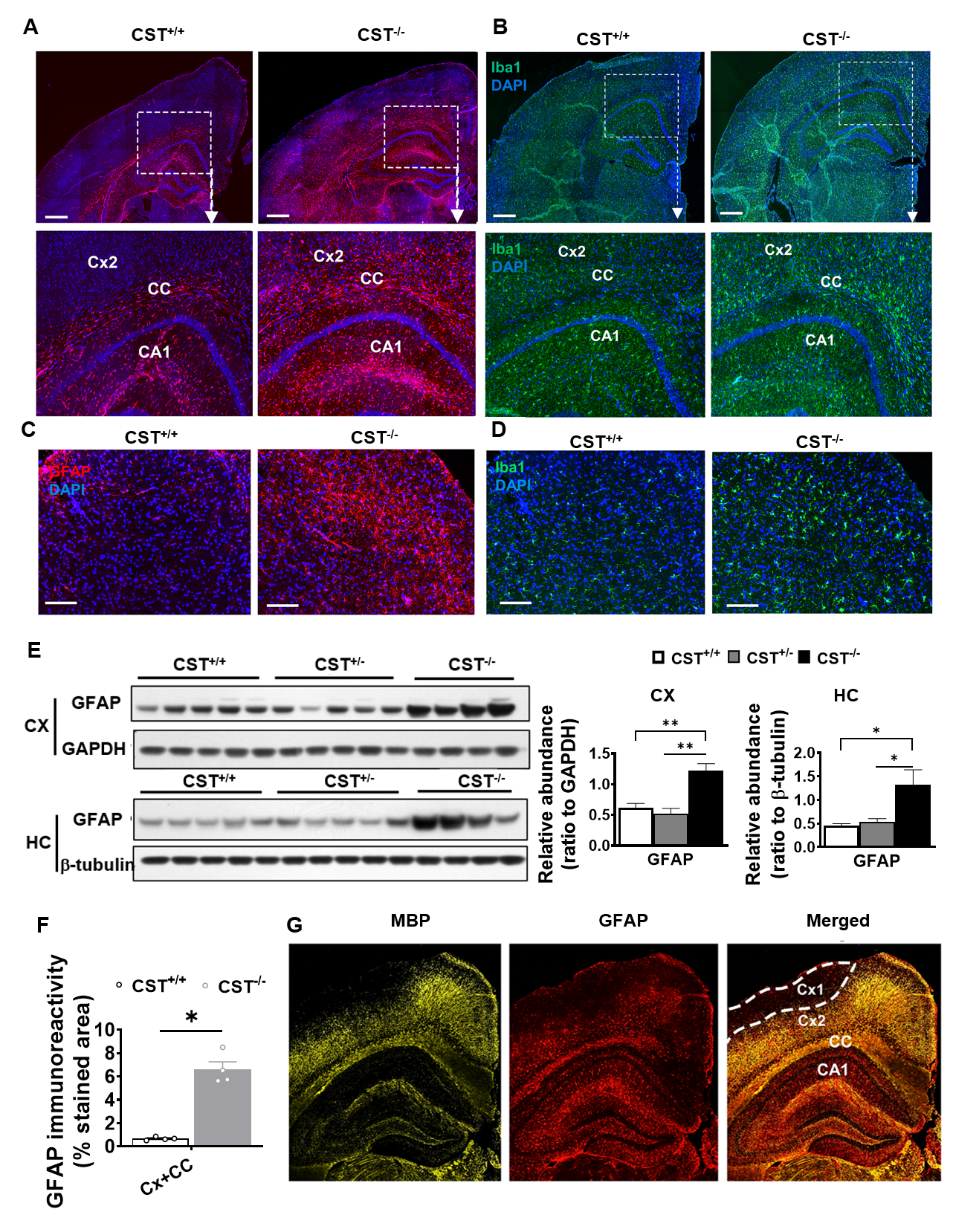


**Fig. S7. Complete ST deletion lead to marked astrogliosis and microgliosis within myelin-containing CNS regions.** Related to Fig. 5. **(A-D)** Representative immunofluorescence images of CST^+/+^ and CST^-/-^ mice using antibodies against GFAP (red) and Iba1 (green) in brain (**A,B**) and brain stem (**C,D**). Quantification of GFAP IF staining area percentage on (cortex + corpus callosum) was shown in (**F**). **(E)** NP40 homogenate supernatants from cortex (CX) and hippocampus (HC) were immunoblotted using antibodies against GFAP and GAPDH/β-tubulin. Relative expression levels of GFAP were quantified and plotted as a ratio to GAPDH/β-tubulin. Two-tailed unpaired t-Test, n=4-5. (**F)** GFAP relative expression on (cortex + corpus callosum) was quantified and plotted as a IF staining area percentage**.** (**G**)Co-staining of GFAP (red) and myelin basic protein (MBP, yellow) in brain of CST^-/-^ mice. Cx: cortex (Cx1: outer cortex without enriched sulfatide; Cx2: inner cortex with enriched sulfatide); CC: corpus callosum; CA1: Cornu Ammonis 1 region of the hippocampus; DG: hippocampal dentate gyrus. Scale bar: 500 μm (**A,B**) and 200 μm (**C,D**). *p < 0.05, **p < 0.01. Data represent the mean ± S.E.M.


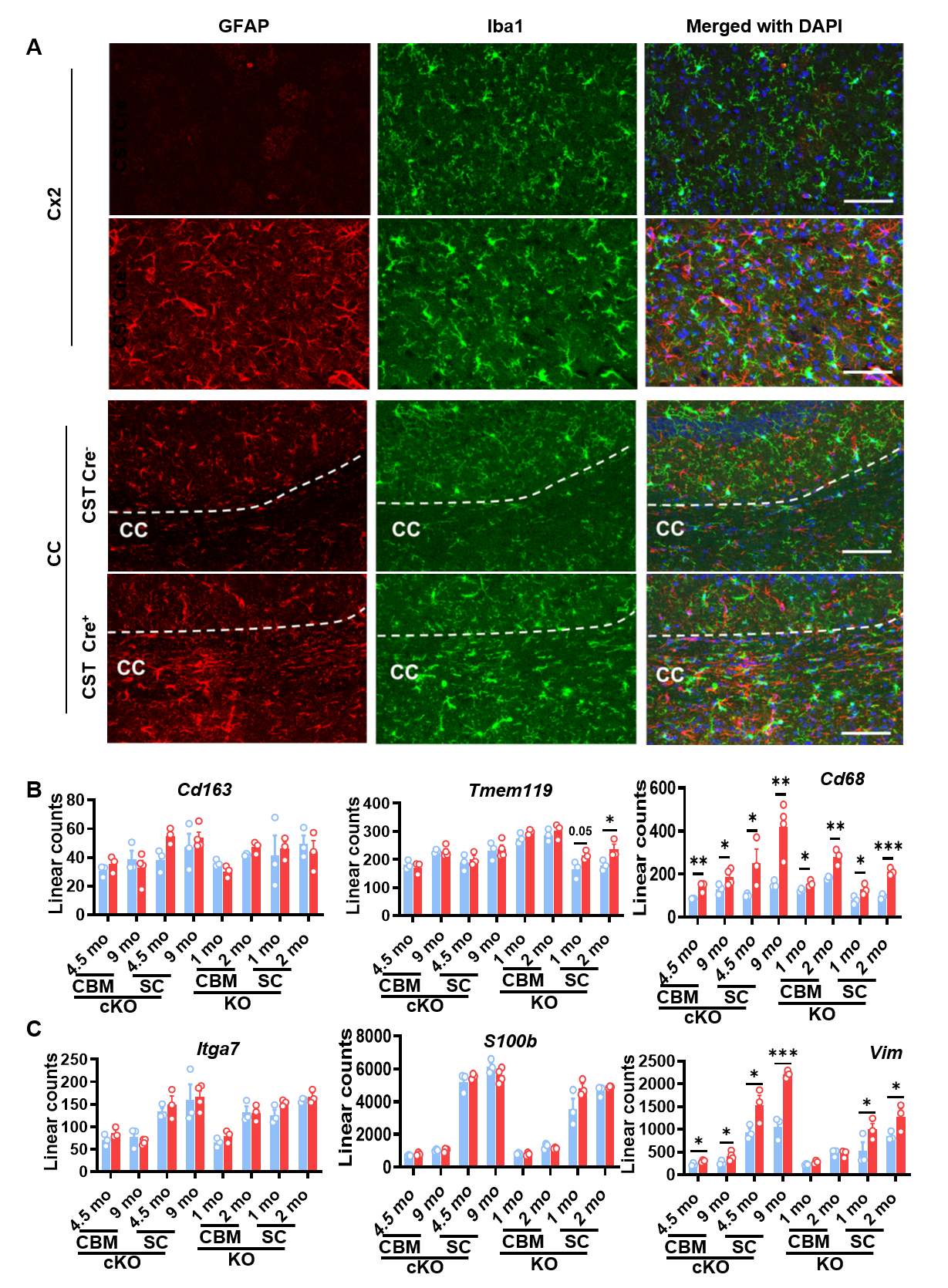


**Fig. S8 The reactive astrocytes and microglia in sulfatide deficient brain were hypertrophic without major microglial or astrocyte proliferation.** Related to Fig. 5. **(A)** Representative immunofluorescence images from GFAP and Iba1 antibody co-staining in the brain shows hypertrophic reactive astrocyte and microglia in cortex and corpus callosum. Cx2: inner cortex; CC: corpus callosum. (**B)** Three microglial-enriched genes are shown. (**C)** Three astrocyte-enriched genes are shown. *p < 0.05, **p < 0.01. Data represent the mean ± S.E.M.


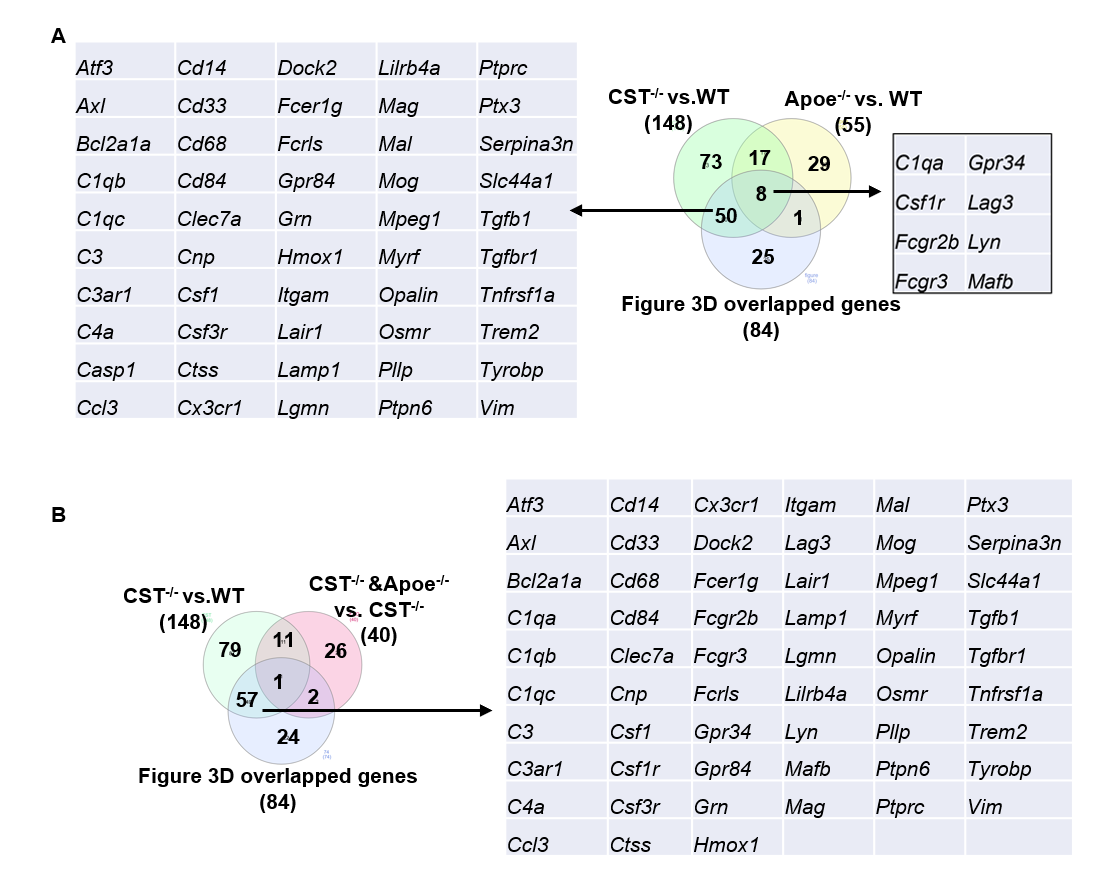


**Fig. S9. (A)** Venn diagrams showing the number of specific and shared upregulated DEGs from ApoE^-/-^ vs.WT, CST^-/-^ vs.WT and the DEGs listed in Fig. 3C. 50 DEGs of the 84 DEGs listed in Fig. 3C that were altered in both CST cKO and KO mice shared only with CST^-/-^ but not ApoE^-/-^, and only eight DEGs of 84 DEGs listed in Fig. 3C were shared amongst both of CST^-/-^ and ApoE^-/-^. (**B)** Venn diagrams showing the number of specific and shared upregulated DEGs from CST^-/-^ vs.WT, ApoE^-/-^/CST^-/-^ vs. CST^-/-^ and the DEGs listed in Fig. 3C. Only three DEGs from 84 DEGs listed in Fig. 3C were shared with the DEGs from ApoE^-/-^/CST^-/-^ vs. CST^-/-^, and 57 DEGs shared in the DEGs from CST^-/-^ vs.WT.


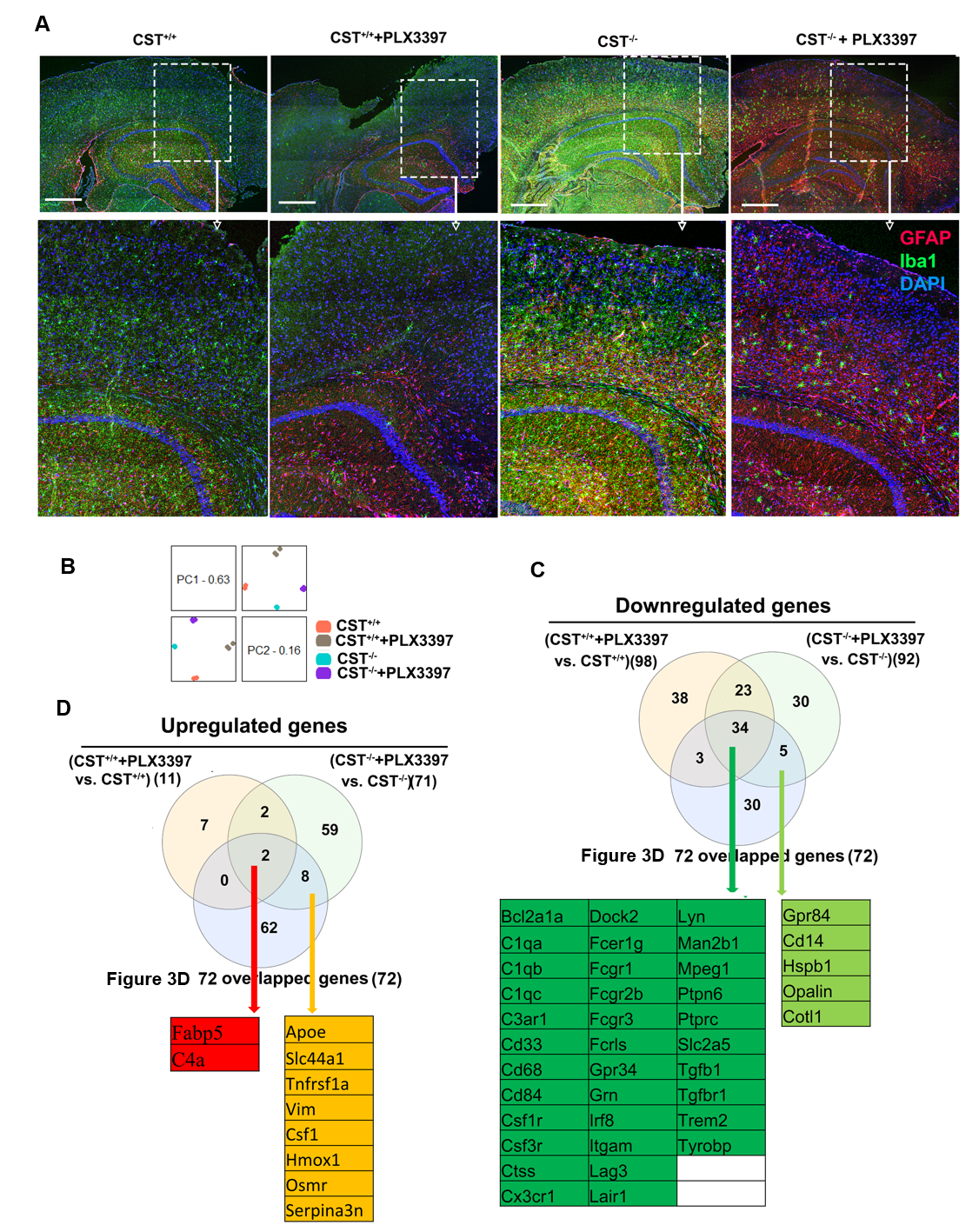


**Fig. S10. Astrogliosis is not secondary to but independent of microgliosis in sulfatide deficient mice.** Related to Fig. 7. **(A)** Representative merged immunofluorescence images stained as described in Fig. 7; Iba-1 (green), GFAP (red) and DAPI (blue). (**B)** PCA from four RNA datasets of 3-mo-old CST KO mice with or without PLX3397 treatment using the NanoString mouse neuroinflammation panel. (**C)** Venn diagrams showing the number of specific and shared downregulated DEGs. (**D)** Venn diagrams showing the number of specific and shared upregulated DEGs. Scale bar: 100 μm.
