# Supplementary figures and images for: Adult-onset CNS myelin sulfatide deficiency is sufficient to cause Alzheimer’s disease-like neuroinflammation and cognitive impairment
Source: Mol Neurodegener. 2021 Sep 15;16:64. doi: 10.1186/s13024-021-00488-7 (PMC8442347; doi:10.1186/s13024-021-00488-7)

Fig.1 F

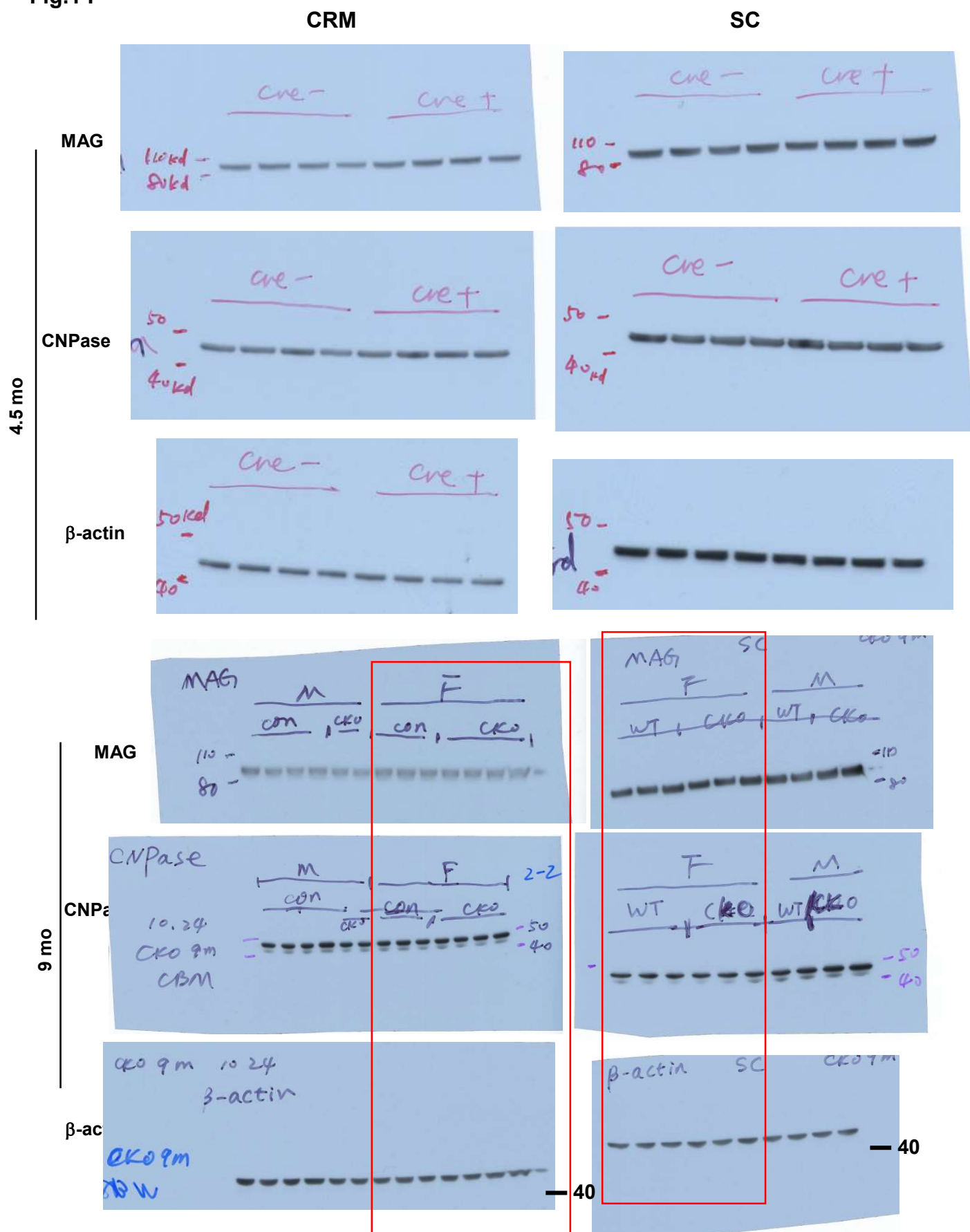

Fig.3 E

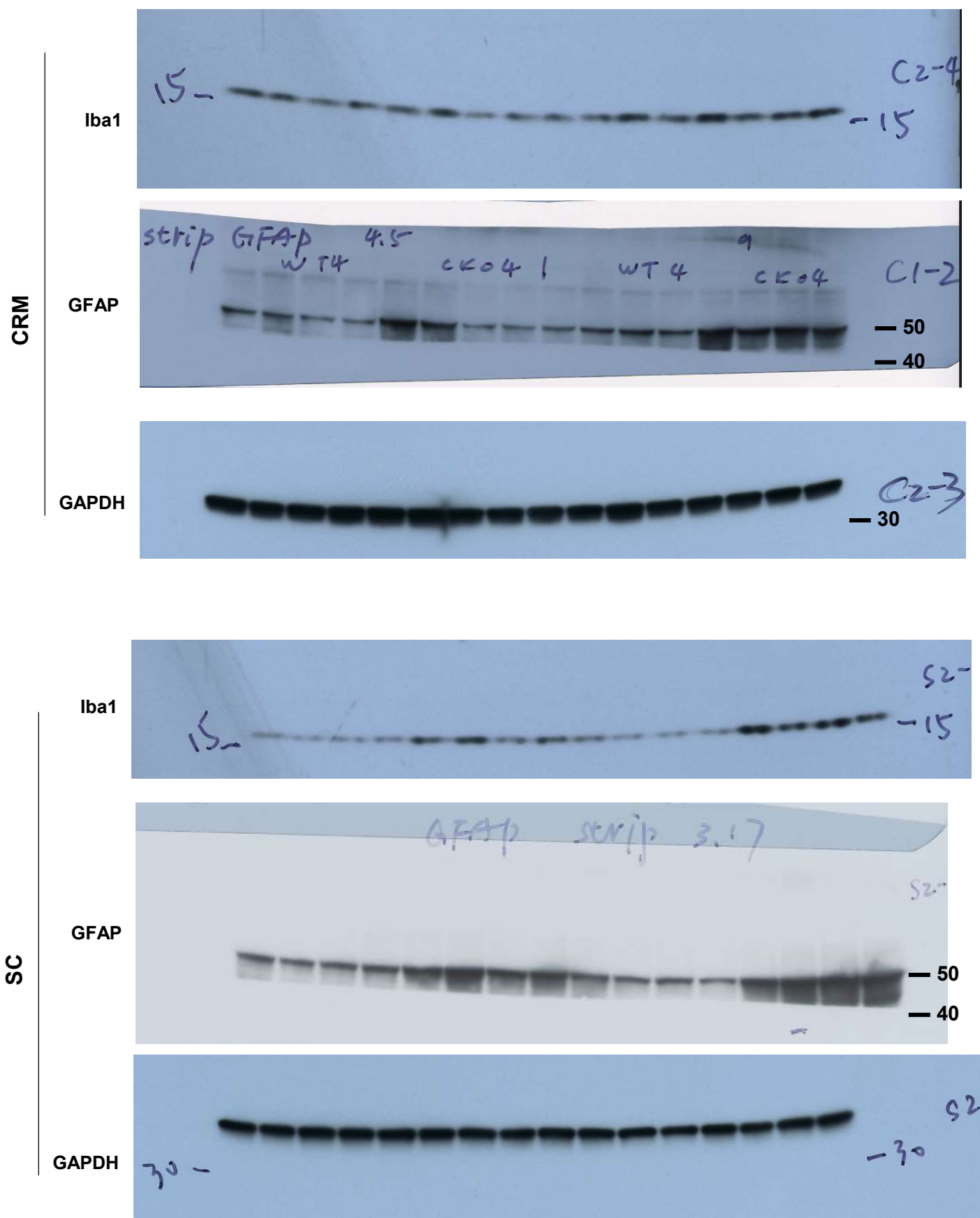

Fig.7 E

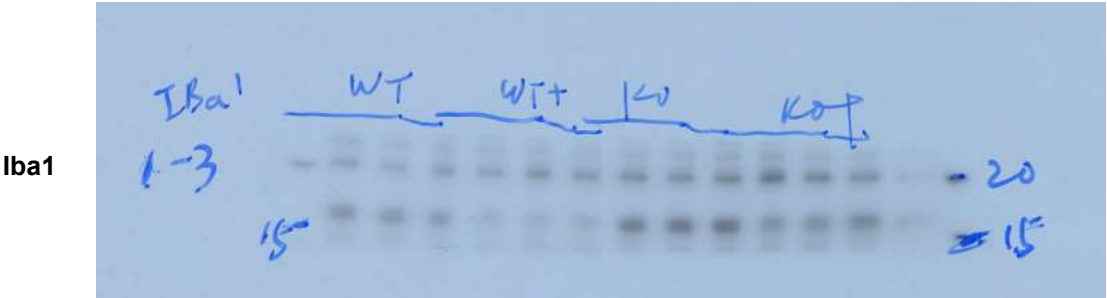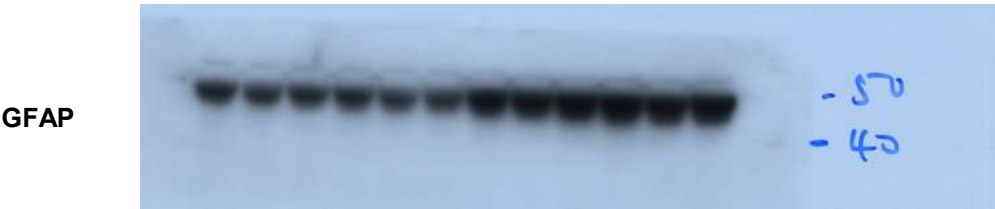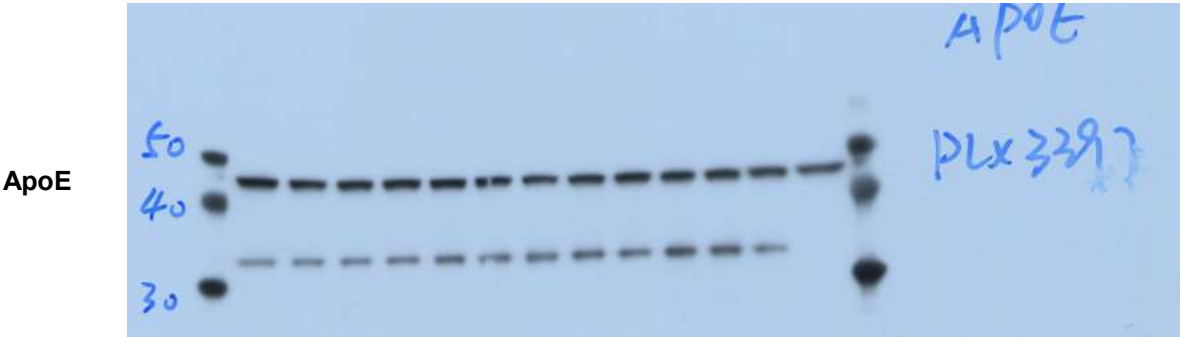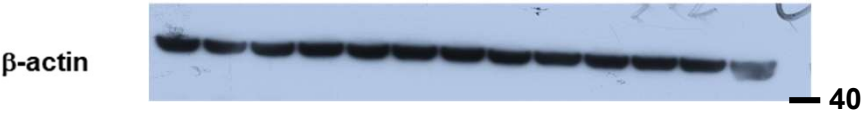

Fig.8B-1

CRM

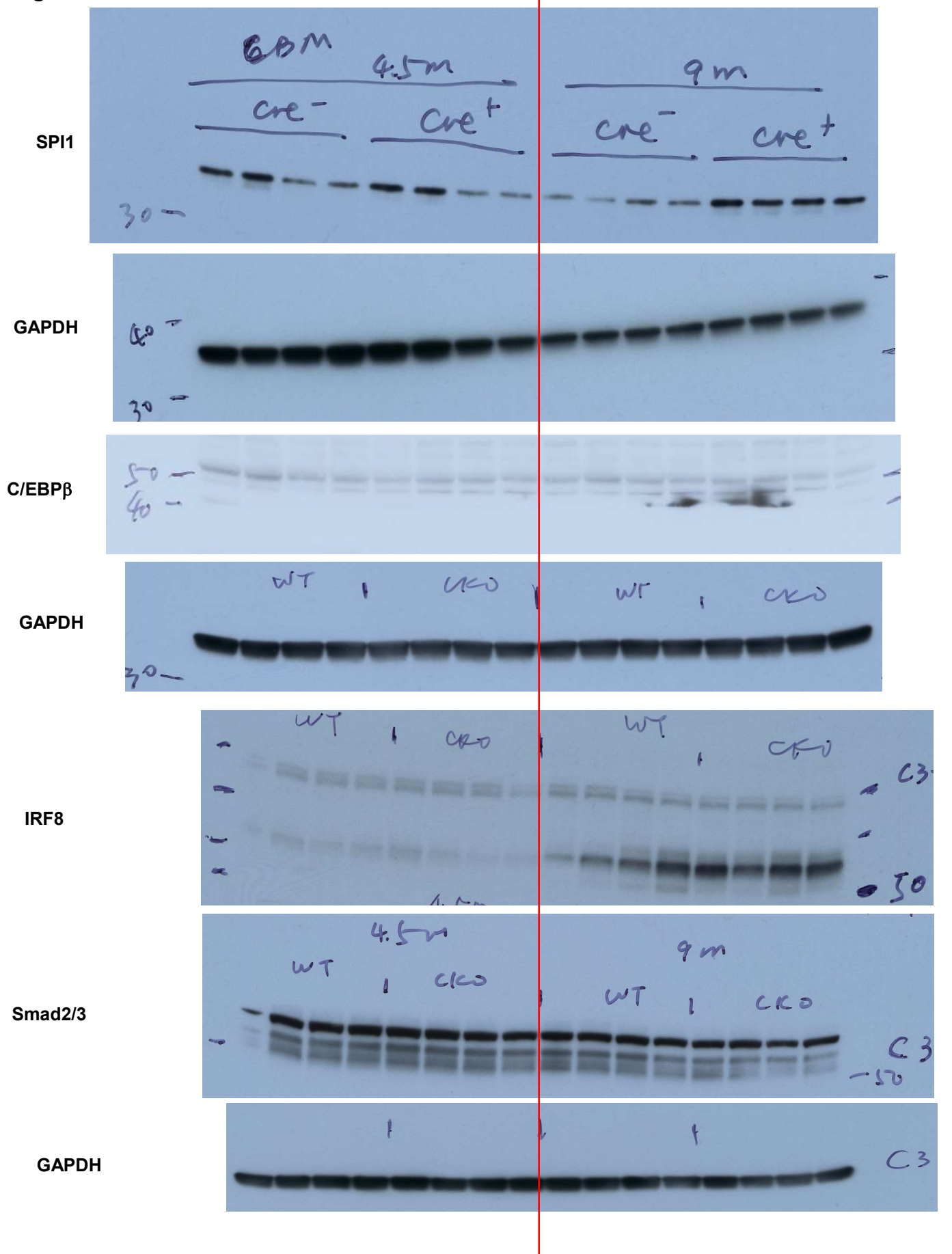

Fig.8B-2

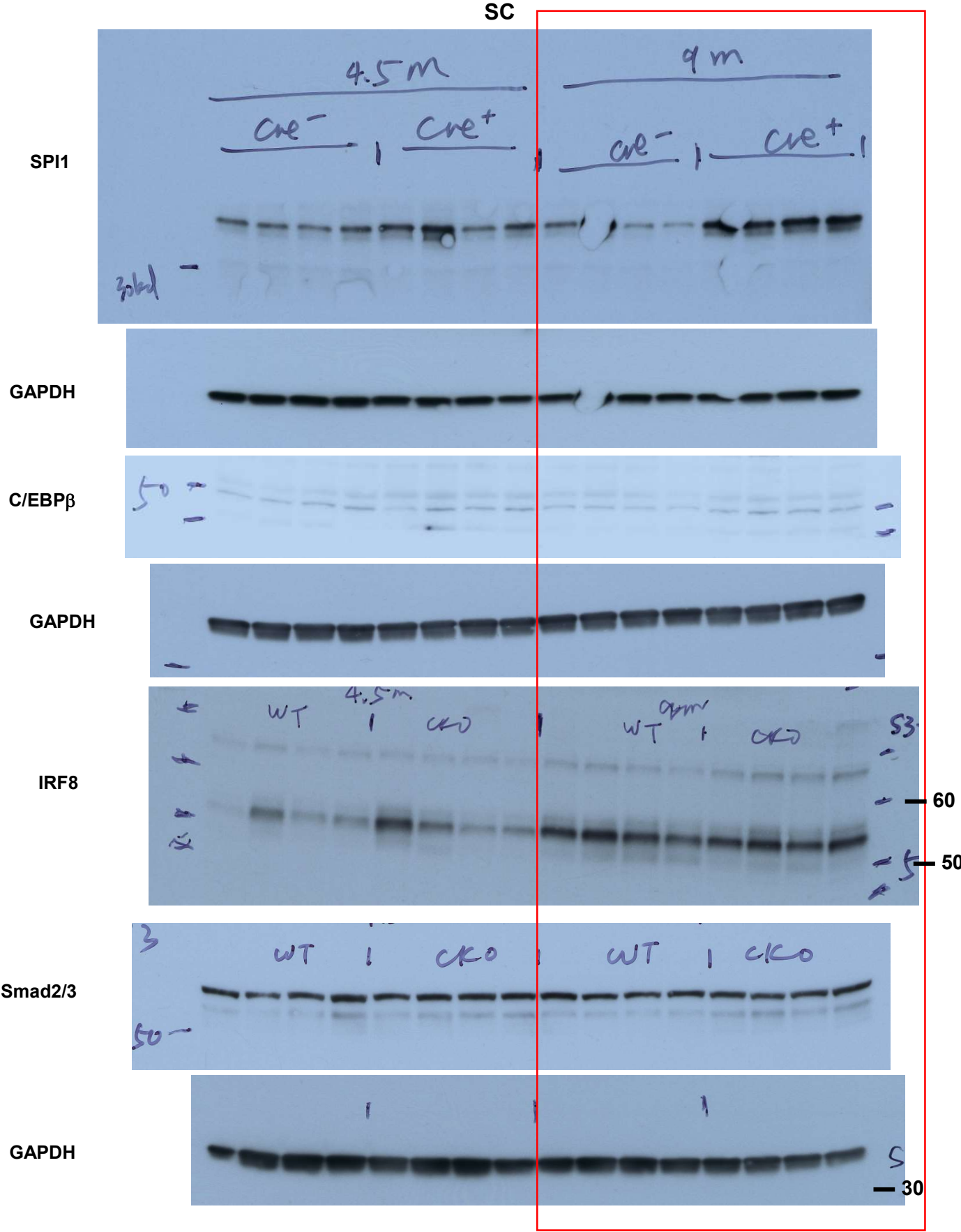

Fig.8B-3

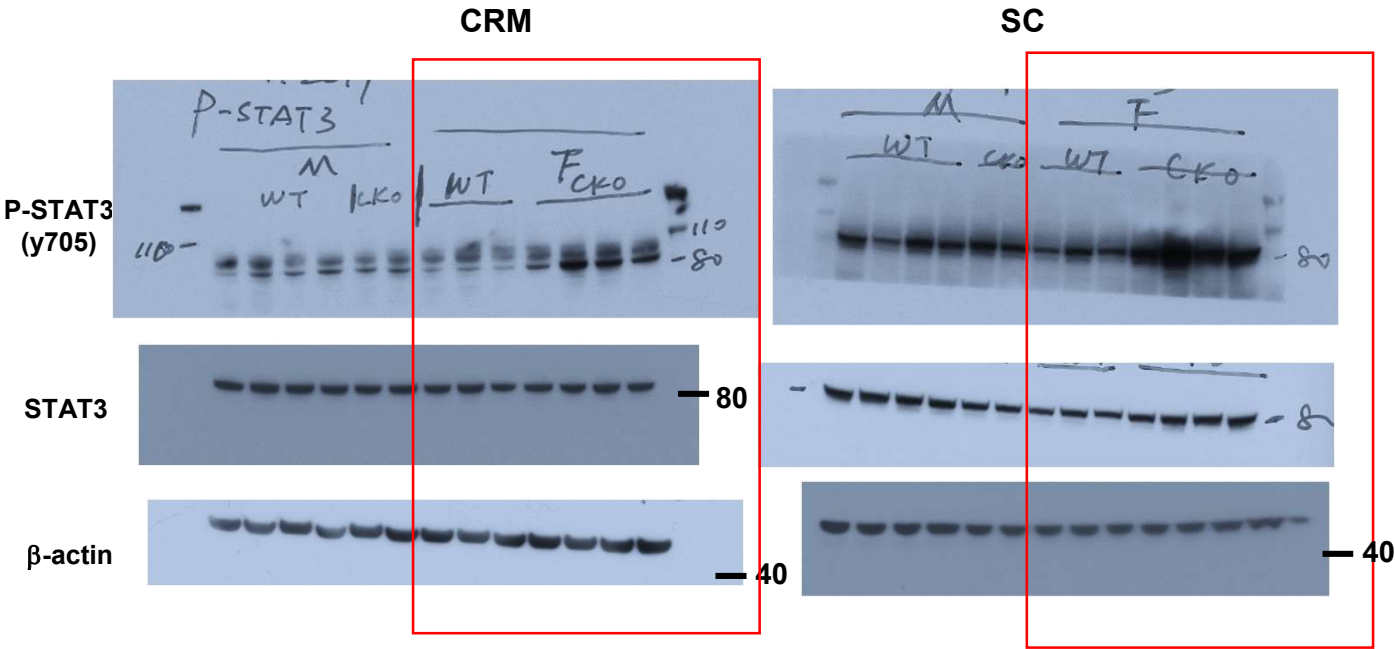

Fig.8D

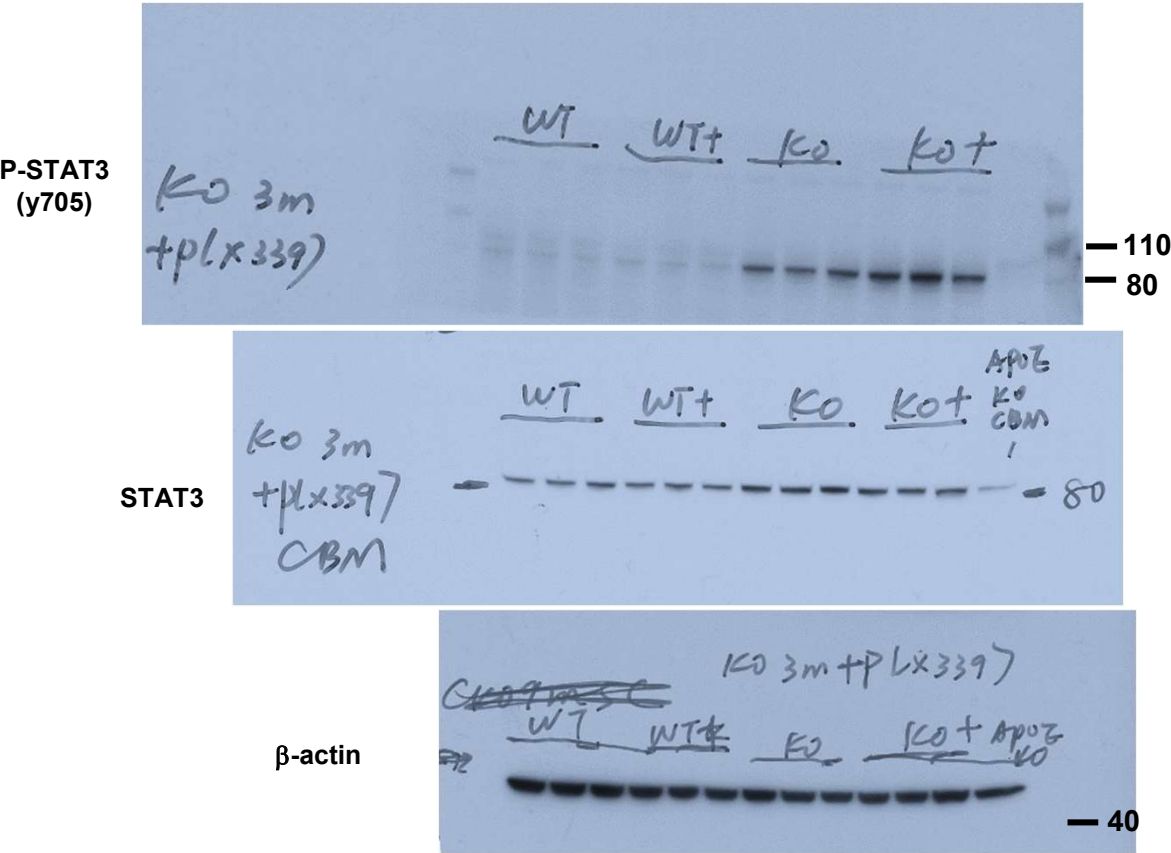

Sup Fig.6A

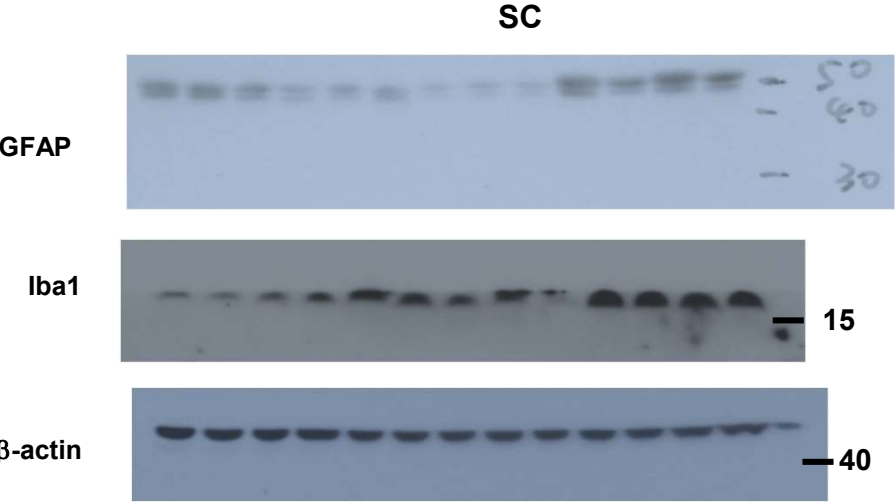

Sup Fig.7E

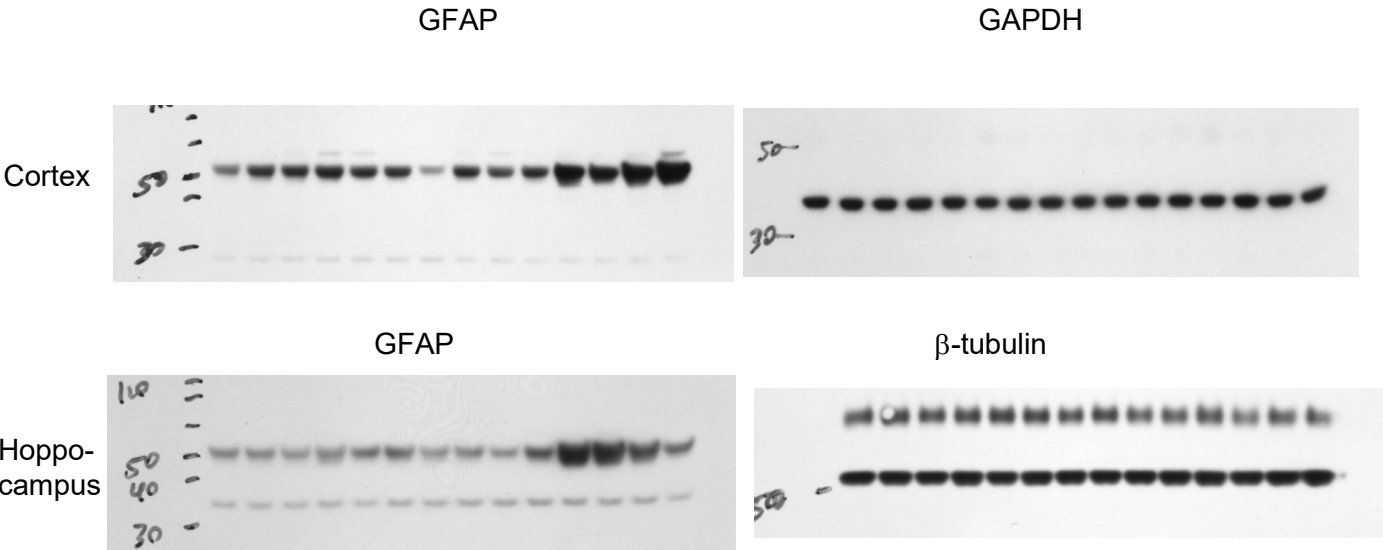

Supplement: Supplementary file 2 — Additional file 2: Original western blot figures. [file 13024_2021_488_MOESM2_ESM.pdf]
